# Cell Membrane Hybrid Lipid Nanovesicles Enhance Innate Immunity for Synergistic Immunotherapy by Promoting Immunogenic Cell Death and cGAS Activation

**DOI:** 10.34133/bmr.0038

**Published:** 2024-07-02

**Authors:** Ruijie Qian, Yawen Guo, Ruihua Wang, Shuai Wang, Xuemei Gao, Ziyang Zhu, Kun Wang, Ke Zhu, Baosong Jia, Yijian Chen, Zhiyu Wang, Jianzhuang Ren, Xuhua Duan, Xinwei Han

**Affiliations:** ^1^ Department of Interventional Radiology, The First Affiliated Hospital of Zhengzhou University, Zhengzhou, China.; ^2^ Department of Immuno-Oncology, The Fourth Hospital of Hebei Medical University, Shijiazhuang, Hebei, China.; ^3^ Department of Nuclear Medicine, The First Affiliated Hospital, College of Medicine, Henan Medical Key Laboratory of Molecular Imaging, Zhengzhou University, Jianshe East Road, Zhengzhou 450052, Henan, China.; ^4^ Department of Medical Technology, Nanyang Medical College, Nanyang 473000, Henan, China.; ^5^ Department of Nuclear Medicine, Sichuan Provincial People’s Hospital, Chengdu, Sichuan 610072, China.; ^6^ Department of Nuclear Medicine, Shanghai East Hospital, School of Medicine, Tongji University, Shanghai 200120, China.; ^7^ Department of Cardiology, Shanghai East Hospital, School of Medicine, Tongji University, Shanghai 200120, China.; ^8^ Department of Breast and Thyroid Surgery, The Second People’s Hospital of Lianyungang, Lianyungang, China.; ^9^ Department of Radiology, Beijing Jingmei Group General Hospital, Beijing, China.

## Abstract

Immunotherapy shows great therapeutic potential for long-term protection against tumor relapse and metastasis. Innate immune sensors, such as cyclic GMP-AMP synthase (cGAS) and stimulator of interferon genes (STING), dissolve DNA and induce type I interferon. Through activation of the cGAS/STING pathway, chemotherapy drugs and reversine (REV) may provide synergetic anti-tumor effects. Here, we prepared drug-loaded cell membrane hybrid lipid nanovesicles (LEVs) (designated LEV@DOX@REV) by fusion of cell membranes, phospholipids, doxorubicin (DOX), and REV, to realize accurate delivery to tumors and chemo-immunotherapy. The cell membranes of LEVs confer “homing” abilities. DOX can induce immunogenic cell death as a result of its specific immunomodulatory effects, which promotes the maturation of immune cells and improves the microenvironment of the immune system. REV is proven to efficiently activate cGAS/STING signaling, thereby enhancing the immune system. The antitumor efficacy of LEV@DOX@REV was evaluated in a 4T1 subcutaneous tumor xenograft model, a distant metastatic tumor model, and a liver metastatic tumor model. LEV@DOX@REV facilitated the infiltration of cytotoxic T lymphocytes within tumors, increased the secretion of proinflammatory cytokines, and modified the tumor microenvironment. In conclusion, LEV@DOX@REV displayed favorable antitumor effects and extended the survival of tumor-bearing mice. We therefore successfully developed nanoparticles capable of enhancing immune activation that have potential therapeutic applications for cancer immunotherapy.

## Introduction

Breast cancer is the tumor with the highest incidence in women; 287,000 women were estimated to be diagnosed with breast cancer in 2022 [1]. Breast cancer patients with distant metastases have a poor prognosis despite advances in early detection and treatment, and are at high risk of drug resistance and tumor recurrence [2]. Immunotherapy emerged as a novel treatment for breast cancer, and its use together with chemotherapy has been approved for the first-line treatment of programmed cell death-1 ligand-overexpressing breast cancer [3]. However, the sensitivity of patients to immune response inhibitors is limited (~20% to 30%). Therefore, improving the response to immunotherapy in breast cancer patients is an issue that requires urgent attention.

Chemotherapy has long been the preferred modality of treatment for cancer. However, there are many shortcomings of conventional chemotherapy, such as drug resistance and side effects. Extensive research has shown that the combination of immunotherapy and chemotherapy could synergistically improve antitumor efficacy [4]. The cyclic GMP-AMP synthase (cGAS)/stimulator of interferon genes (STING) pathway, which plays a key role in innate immunity, is emerging as a promising therapeutic method for cancer [5]. In brief, the cGAS/STING pathway can be activated by cytosolic DNA, and this pathway can promote the production of interferon (IFN)-I and the secretion of pro-inflammatory cytokines to activate the innate immune response [6]. Chemotherapy can be enhanced by cGAS/STING-induced immunity, according to recent studies [5,6]. In addition, reversine (REV) has been proven to be efficient at activating cGAS/STING signaling in breast cancer. Combining chemotherapy drugs with REV could enhance antitumor efficacy through the initiation of the cGAS/STING pathway. Doxorubicin (DOX) can induce immunogenic cell death (ICD) due to its specific immunomodulatory effects, which promotes the maturation of immune cells and improves the microenvironment of the immune system [7,8]. As a result, the ICD process is characterized by the up-regulation of calreticulin (CRT) from tumor cells, causing dendritic cells (DCs) to recognize CRT, thus promoting antitumor immunity [9]. In addition to stimulating antigen-presenting DCs, high mobility group box 1 protein (HMGB1) in tumor cells secretes adenosine triphosphate (ATP) to recruit DCs to tumors [10]. Thus, ICD induction is an efficient immune therapy for the prevention of tumor invasion and metastasis [11]. Cancer cells would undergo ICD when DOX-mediated chemotherapy was applied, accompanied by the release of tumor antigens, which would recruit host DCs. After antigen uptake and processing, DCs developed mature characteristics, presenting antigenic peptides to naive T cells to elicit antitumor immunity. Cancer cells would be lysed by activated CD8 T cells infiltrating tumor tissue [12].

However, the activation of cGAS/STING in cancer cells remains a major challenge. The main problem with STING agonists in clinical practice is the possible treatment-related side effects associated with cytokine induction, especially following systemic administration where a higher dosage is needed to achieve therapeutic efficacy. Systemic delivery of tumor-targeting STING agonists may overcome such limitations [13]. Nanomedicines have been developed in immunotherapy, including therapeutic strategies for cGAS/STING signaling [14,15]. As a result of their drug-loading capacity, excellent biocompatibility, and tumor-targeting capabilities, tumor-derived exosomes are thought to be promising drug carriers [16–18]. However, a variety of studies have shown that the specific features of tumor-derived exosomes involved in tumor metastasis might be associated with the mortality rate of patients. Certain tumor-derived exosome (TEX) proteins on TEXs (such as ITGαvβ5 and ITGα6β1) could mediate tumor metastasis [19]. They are also limited by the difficulty of isolation and purification, as well as insufficient cargo encapsulation when it comes to clinical translatability and pharmaceutical application [20]. Synthetic nanocarriers have many advantages over natural nanocarriers, including controllable size, high drug loading efficiency, intelligent drug release capability, and facile surface modification properties. To address this issue, it is expected that a new technique for extracting the cellular membrane might extend the use of tumor-derived exosomes. Cell membrane-camouflaged technology has found wide applications in a variety of cellular biomimetic nanoparticles, such as cancer cells and red blood cells [21]. Briefly, the cell membrane is obtained by removing the cellular contents, and then the remaining membrane is combined with a flexible base material to produce the nanoparticles [22].

**Fig. 1.**
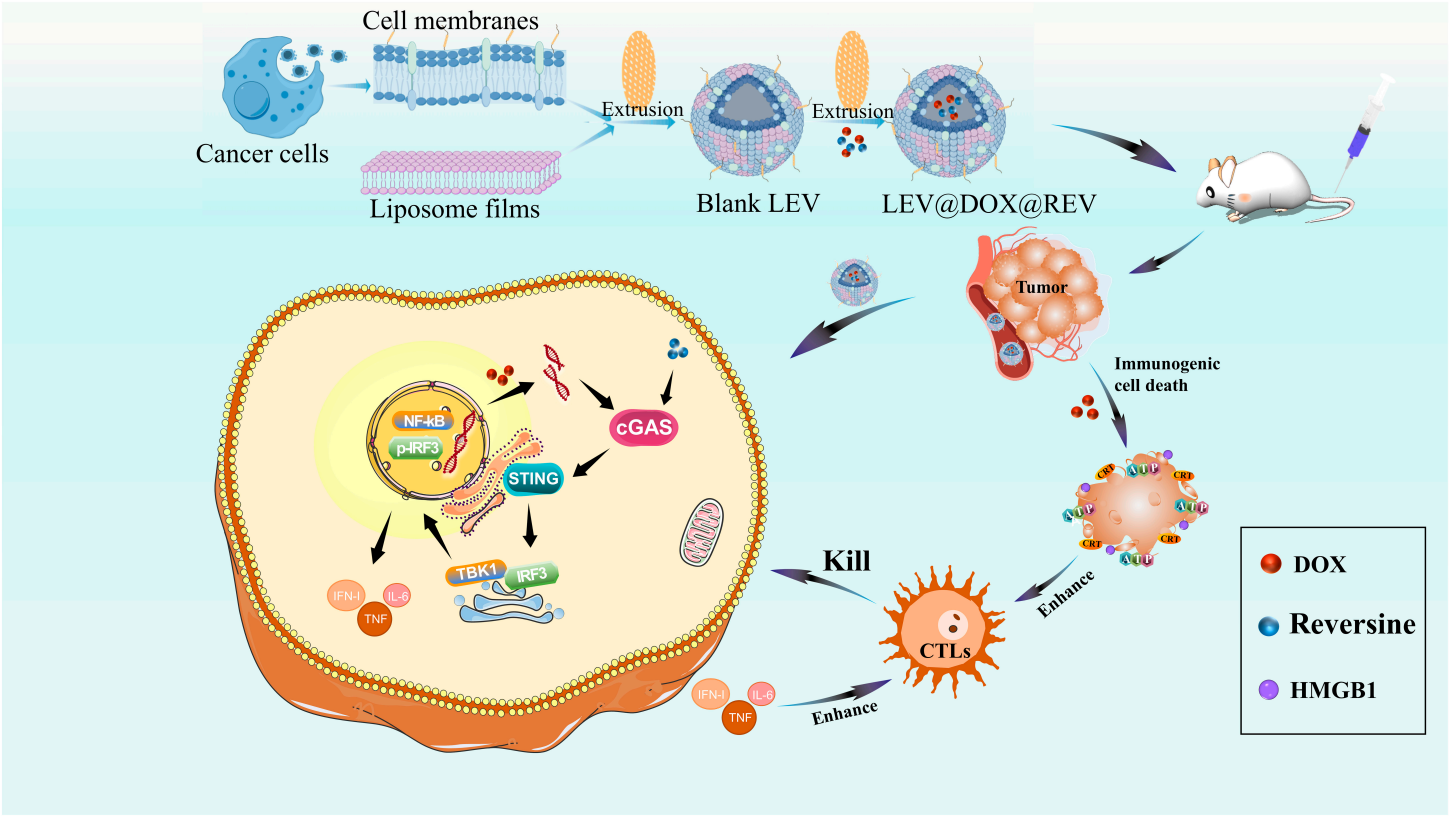
Schematic diagram of LEV@DOX@REV used for combined therapy that functions via enhancing the cGAS/STING pathway and ICD.

In this study, we designed novel nanovesicles fabricated by hybridization of tumor cell membranes and phospholipids for DOX and REV delivery, designated LEV@DOX@REV. It is expected that the LEV@DOX@REV membrane derived from breast cancer 4T1 cells will inherit the tumor-targeting ability of their parent tumor cells. A hybrid lipid-cell membrane may improve safety and preserve tumor-targeting ability, making it an ideal therapeutic agent for cancer. As such, LEV@DOX@REV was expected to promote DC maturation and increase T cell infiltration. Additionally, activation of the cGAS/STING downstream pathway leads to an increase in IFN-I production and TNF production, the 2 factors combined that lead to an anti-tumor response (Fig. 1).

## Materials and Methods

### Cell culture and animals

4T1 cells (mouse breast cancer cell line) were cultured in RPMI-1640 medium (Gibco) with 10% fetal bovine serum (Gibco) in a humidified atmosphere at 37 °C containing 5% CO_2_.

### Preparation of liposomes or drug-loaded liposomes

Liposomes were prepared by the membrane hydration method, as previously reported [23]. Briefly, lipids with a ratio of DSPC/DSPE-PEG_2000_/Chol of 90:10:4 (mol/mol/mol) were dissolved in chloroform for 30 min. Subsequently, a rotary evaporator was used to eliminate the organic solvent at 55 °C under vacuum conditions. The lipid film was washed with 10 ml of PBS for 2 h at 65 °C, and then the samples were subjected to ultrasound for 10 min and subsequently filtered 3 times through a polycarbonate membrane with a pore size of 100 nm. To prepare DOX and REV-loaded liposomes, DOX and REV were added into the lipid solutions at a drug/lipid ratio of 1:15 (w/w), and the remaining procedures were consistent with the steps for blank liposome preparation. After filtering through a polycarbonate membrane, the samples were added to 3,500-kDa dialysis bags and dialyzed in PBS for 8 h to remove the free drugs.

Exosome mimetic (EM) was prepared according to a previously reported method [24]. 4T1 cells were obtained with trypsin and resuspended in a hypotonic solution (ddH_2_O:PBS = 3:1). Broken 4T1 cells were centrifuged for 10 min at 700×*g*, then again for 30 min at 14,000×*g*. Finally, the plasma membrane precipitate was collected for further investigation and the 4T1 cell membrane solutions were physically extruded through a 0.1-μm polycarbonate membrane 11 times. DSPE-PEG_2000_-Indocyanine Green (ICG) was incubated with LEV@DOX@REV for 30 min at 37 °C to form ICG-LEV@DOX@REV. The sample solutions were passed through centrifugal filter devices (100 kDa molecular weight, AmiconUltra-15) to remove the unreacted reagents.

### Preparation of hybrid lipid nanovesicles or drug-loaded LEVs

Lipid nanovesicles (LEVs) were synthesized by the thin layer evaporation method and extrusion process. Briefly, lipids with a ratio of DSPC/DSPE-PEG_2000_/Chol of 90:10:4 (mol/mol/mol) were dissolved in chloroform for 30 min, and organic solvents were removed to form a thin film by rotary evaporator at 55 °C under vacuum conditions. Films were hydrated with 4T1 cancer cell membrane dispersed in PBS to assemble LEVs by heating and vortexing at 45 °C for 20 min. Conventional liposomes were prepared as described above with PBS alone. Then, the solutions with or without drugs (DOX: 10 μg/ml; REV: 10 μg/ml) were physically squeezed through a 0.1-μm polycarbonate membrane 11 times. DSPE-PEG_2000_-ICG were incubated with LEV@DOX@REV for 30 min at 37 °C to form ICG-LEV@DOX@REV. The sample solutions were passed through centrifugal filter devices (100 kDa molecular weight, AmiconUltra-15) to remove the unreacted reagents.

To explore the ratio of cell membranes and liposomes in LEVs, cell membranes and liposome films were labeled with DiO (10 μg/ml) and DiI (10 μg/ml), respectively. LEVs were prepared with different proportions of liposomes and cell membranes. Then, a microplate reader was used for fluorescence resonance energy transfer (FRET) studies. The fluorescence emission spectra were acquired using excitation at 440 nm and were recorded from 470 to 750 nm.

Particle size and zeta potential distributions were determined by a Zetasizer Nano ZS (Malvern Instrument, Malvern, UK). To analyze stability, LEV@DOX@REV solutions were stored at 4 °C. Morphological characteristics were confirmed by transmission electron microscopy (TEM). The representative protein of LEV@DOX@REV was detected by Western blotting (WB).

### Drug loading capacity and drug release

To study the release of DOX and REV from LEV@DOX@REV, EM@DOX@REV, and Lip@DOX@REV, the nanovesicles were immersed in either an endosomal environment (pH 5.0 PBS) or a simulated physical environment (pH 7.0 PBS) in dialysis bags (molecular weight cutoff 100 kDa, Millipore, Germany) at 37 °C. Dissolution media samples were withdrawn for subsequent analysis and new buffer solutions were added at predetermined intervals. Finally, using a microplate reader, the amount of DOX or REV released was quantified. The formula used to calculate drug loading efficiency (DLE) was as follows:

DLE%=weightofdrug/weightofinputdrug×100% (1)

### Stability study

After storing LEV and LEV@DOX@REV in PBS for 7 days at 4 °C, the storage stability was evaluated. The stability of dynamic light scattering (DLS) and the Zeta potential were assessed at various time points.

### WB analysis

The 4T1 cell lysate, LEV@DOX@REV, and EM@DOX@REV were first prepared, and then treated on ice for 20 min with radioimmunoprecipitation assay lysis buffer (R0010, Solarbio) with phenylmethanesulfonyl fluoride (P0100, Solarbio). To extract the proteins, the mixtures were centrifuged at 12,000 rpm for 20 min at 4 °C. The BCA protein assay kit (P0011, Beyotime) was used to determine the protein concentration in the supernatants. Using 10% sodium dodecyl sulfate polyacrylamide gel electrophoresis (SDS-PAGE), equivalent amounts of proteins were separated. The proteins were then transferred onto a polyvinylidene fluoride membrane. The membrane was blocked with 5% skim milk in 1× TBST (Tris-buffered saline with Tween-20) for 1 h and then incubated with anti-CD44 antibody (1:1,000), anti-β-tubulin antibody (1:1,000), anti-GAPDH antibody (1:5,000), and anti-Histone H3 antibody (1:500) overnight on a shaker at 4 °C. After washing 3 times with TBST for 10 min, the membrane was incubated with secondary antibody (1:5,000) at room temperature for 1 h. After further TBST washing, the membrane was incubated with an enhanced chemiluminescence reagent, then visualized using a ChemiDoc XRS Gel image system (Bio-Rad, Hercules, CA, USA). This WB assay was used to analyze the protein levels of cGAS (1:1,000), STING (1:500), p-TBK1 (1:1,000), TBK1 (1:1,000), p-IRF3 (1:1,000), and IRF3 (1:1,000) in 4T1 tumor cells after various treatments. The antibodies used are listed in Table S1.

### Cellular uptake and localization of LEV@DOX@REV

A total of 6 × 10^4^ cells per well were seeded into 24-well plates and incubated for 24 h. Tumor cells were analyzed by flow cytometry (FCM) or fluorescence confocal microscopy after treatment with LEV@DOX@REV (0, 0.2, 0.5, 1, 2, 5, and 10 μg/ml) at various time points (5, 10, 15, 30, and 60 min).

### In vitro cytotoxicity assay

In a typical experiment, 4T1 cells were seeded into 96-well plates (5 × 10^3^ cells per well) and then cultured with PBS, free DOX+REV, Lip@DOX@REV, EM@DOX@REV, or LEV@DOX@REV for 24 h at 37 °C (DOX: 2 μg/ml; REV: 2 μg/ml). LO-2, HEK-293T, and 4T1 cells were also inoculated with PBS, Lip, EM, and LEVs for 24 h at 37 °C. Then, cells were washed and placed in fresh medium. Finally, cell viability was evaluated using a Cell Counting Kit-8 (CCK-8) according to the manufacturer’s protocol.

### In vitro maturation of DCs

DC2.4 cells were seeded into 12-well plates (10^5^ cells/well) and then treated with different drug-pretreated 4T1 cell culture media and incubated for 24 h. Finally, cells were harvested, stained with anti-CD80 and anti-CD86 antibodies, and analyzed using FCM.

### Animal models

The Zhengzhou University Animal Care and Use Committee supervised, guided, and approved all animal studies. A specific-pathogen-free environment was used to raise Balb/c mice (females, ~6 weeks old). Each mouse was subcutaneously inoculated (1 × 10^7^ cells in 100 μl of PBS) in the right upper limb. After the development of a xenograft tumor, the tumor volume was calculated using the formula: length × width^2^ × 0.5. To evaluate the distant tumor growth inhibition effect of LEV@DOX@REV, 4T1 primary tumor models were first established on day −16. After routine treatment on day −8, primary tumors were surgically removed on day 0. Then, on day 0, 1 × 10^7^ 4T1 cells in 100 μl of PBS were inoculated into the distal side of mice to establish a 4T1 distant tumor model. Mice were subsequently monitored for tumor volume (*n* = 8), weight changes (*n* = 8), and survival (*n* = 10).

### In vivo imaging

When the tumor volume reached approximately 100 to 200 mm^3^, ICG (1 μg/mouse)-labeled Lip@DOX@REV, EM@DOX@REV, and LEV@DOX@REV were injected into the tumor-bearing mice. The fluorescence signals of ICG were obtained at different post-injection times (0, 1, 3, 6, 12, 24, and 48 h) by an IVIS system (PerkinElmer Inc.) (ex: 745 nm; filter: 830 nm). At 24 and 48 h after injection, mice were euthanized, and major organs were collected for ex vivo fluorescence imaging.

### ELISA

The HMGB1 ELISA (enzyme-linked immunosorbent assay) kit was used to analyze HMGB1 release. Following 1-day incubation in 24-well plates (6 × 10^4^ cells/well), cells were treated with or without *N*-acetylcysteine (NAC; 5 mM) for 4 h. Then, culture medium was replaced and added with PBS, LEV@DOX, LEV@REV, or LEV@DOX@REV (DOX: 2 μg/ml; REV: 2 μg/ml). The ELISA kit was used to measure the level of HMGB1 in the supernatants, and the ENLITEN ATP Assay System Bioluminescence Detection Kit was used to detect extracellular ATP.

### Therapeutic effect in animals

To investigate the efficacy of LEV@DOX@REV against primary tumors, mice with tumor volumes up to 80 mm^3^ were randomized into 4 groups (*n* = 8 in each group): (a) PBS; (b) free DOX+REV; (c) Lip@DOX@REV; and (d) LEV@DOX@REV (DOX: 4 mg/kg, REV: 4 mg/kg). The formulations were administered intravenously via the tail vein unless stated otherwise. We recorded the tumor volume every 2 days for a period of 19 days. Endpoints included tumor volumes >1,500 mm^3^, ulcerating tumor tissue, mortality, and >15% weight loss. In the study to investigate the distant tumor growth inhibition effect of LEV@DOX@REV, treatment regimens were provided as described previously (DOX: 4 mg/kg, REV: 4 mg/kg).

### In vivo metastasis assay

For the liver metastasis model, 4T1 cells were injected into the spleens of mice. On day −8, 4T1 subcutaneous graft tumor models were constructed. On day −1, 4T1 tumor-bearing mice were randomly divided into the PBS and LEV@DOX@REV groups. On day 0, 1×10^5^ 4T1 cells in 40 μl of PBS were injected into the spleens to form liver metastases. Then, mice were monitored. On day 10, mice were euthanized to analyze their liver metastases (DOX: 4 mg/kg, REV: 4 mg/kg).

### Biosafety evaluation

Histological, hematological, and biochemical examinations were conducted to assess biosafety in vivo. The drugs were systemically administered through a single tail vain injection. At the experimental endpoint, several major organs were excised and stained for histological analysis. A hematology analyzer (BC-2800Vet, Mindray, China) was used to analyze whole blood samples. Using an automatic biochemistry analyzer (Chemray240, China), serum was separated from whole blood samples and assessed for hepatotoxicity and nephrotoxicity. Hepatic function indicators included aspartate aminotransferase (AST), alanine aminotransferase (ALT), and alkaline phosphatase (ALP). Renal function indicators included blood urea nitrogen (BUN) and creatinine (CREA).

### Quantitative real-time PCR

4T1 cells were seeded into 6-well plates and incubated for 24 h. PBS, LEV@DOX, LEV@REV, or LEV@DOX@REV was then added to each well. As directed by the manufacturer, total RNA was extracted from cell pellets using TRIzon reagent (Invitrogen, USA). Reverse transcription was performed using 1 μg of total RNA and the HiFiScript 1st strand cDNA synthesis kit. Then, 2×Universal SYBR GREEN qPCR Super Mix (UE, Suzhou, China) was used to measure the expression levels of the target genes. The qPCR program involves an initial denaturation at 95 °C for 2 min (step 1), then amplifications were carried out with 40 cycles at a melting temperature of 95 °C for 5 s (step 2) and an annealing temperature of 60 °C for 30 s (step 3), followed by melt curve analysis. To normalize mRNA levels, GAPDH mRNA levels were used as a reference. The primers used are listed in Table S2.

### Study of the infiltration of immune cells

The harvested tumors and spleens of mice were cut into small pieces and digested to obtain single-cell suspensions at 37 °C for 2 h in serum-free medium. The cells were washed in PBS and centrifuged at 1,500 rpm for 5 min at 4 °C and treated with red blood cell lysis buffer for 5 min to lyse red blood cells. CD8+ or CD4+ T cells in tumors were stained with anti-CD3 antibody (1:100), anti-CD4 antibody (1:100), or anti-CD8 antibody (1:100) to test T cell activation. Macrophages in tumors were stained with anti-F4/80 antibody (1:100), anti-CD86 antibody (1:100), or anti-CD206 antibody (1:100) to test macrophage polarization. Memory T cells in spleens were stained with anti-CD8a antibody (1:100), anti-CD44 antibody (1:100), or anti-CD62L antibody (1:100). DCs in spleens were stained with anti-CD86 antibody (1:100) or anti-CD80 antibody (1:100) to test DCs maturation. The antibodies used are listed in Table S1. Finally, cells were washed with PBS and harvested for FCM analysis.

### Statistical analysis

Data are presented as the mean ± standard deviation (SD). Statistical analysis was performed using GraphPad Prism 9 software. A *P* value < 0.05 was regarded as statistically significant.

## Results

**Fig. 2.**
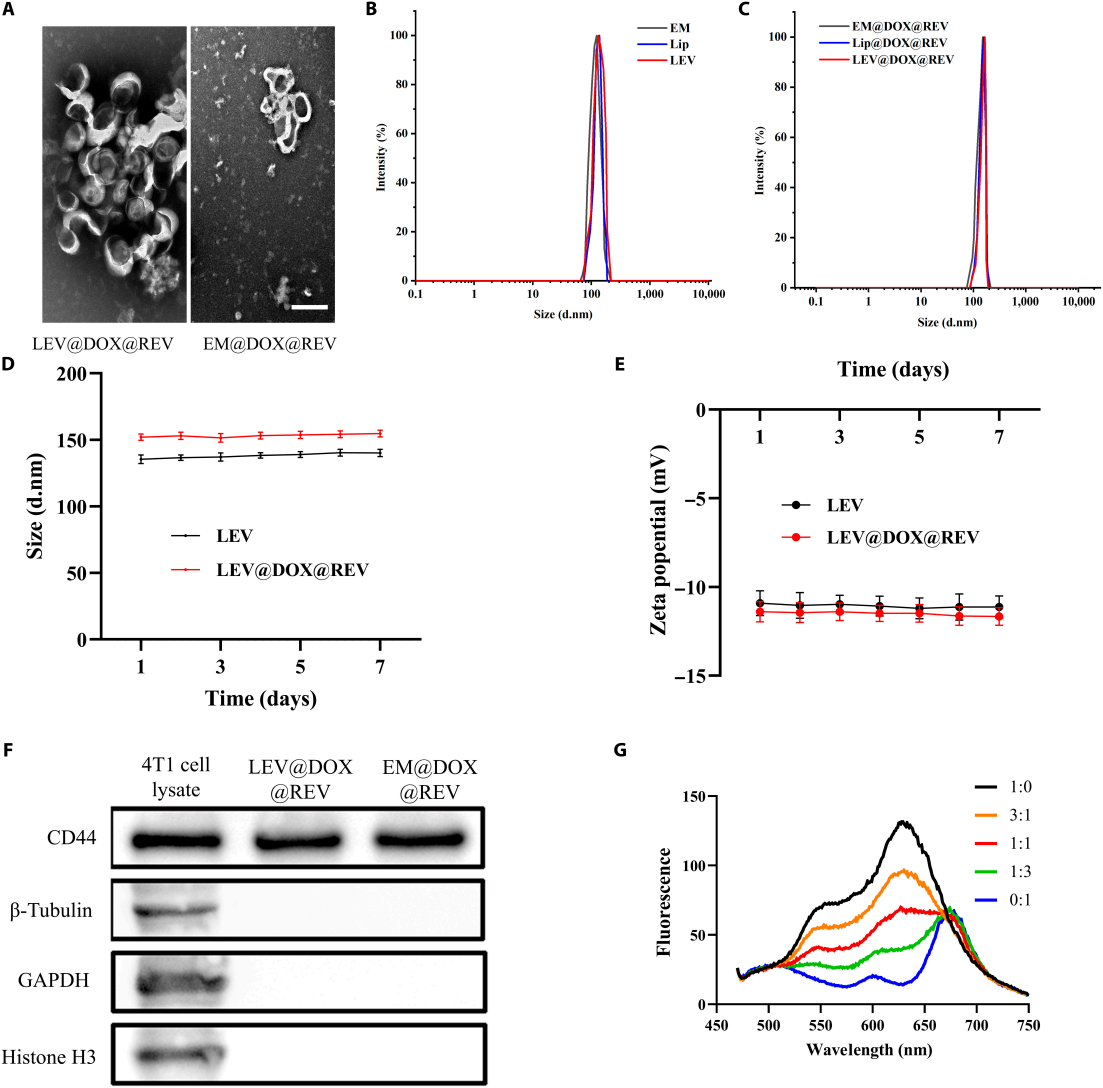
Preparation and characterization of LEV@DOX@REV. (A) TEM image of LEV@DOX@REV. (B) DLS was used to measure the hydrodynamic diameter of LEVs before (C) and after (D) drug loading. (E) The zeta potential of LEV@DOX@REV was assessed after 7 days. (F) Western blotting analysis. Samples were run at equal protein concentration and immunostained against membrane markers. (G) The optimal ratio of cell membranes and liposome film membranes in LEVs, as analyzed by FRET. Cell membranes were labeled with DiO and liposome film membranes were labeled with DiI.

TEM displayed the typical cup-shaped morphology of LEV@DOX@REV (Fig. 2A). DLS showed no significant change in the particle size before or after drug loading (Fig. 2B and C). LEV@DOX@REV slightly changed in size from 152.0 ± 2.4 nm on day 1 to 154.7 ± 2.6 nm on day 7; the Zeta potential of LEV@DOX@REV also changed slightly from −11.4 ± 0.6 mV on day 1 to −11.7 ± 0.5 mV on day 7, indicating the excellent stability of the nanoplatform (Fig. 2D and E). The results demonstrated that LEV@DOX@REV and EM@DOX@REV presented similar protein profiles (Fig. 2F). In LEV@DOX@REV and EM@DOX@REV, membrane-specific markers were inherited and detectable from 4T1 cells, while GAPDH and nuclear protein markers (Histone H3) were almost undetectable. To determine the ratio of these components to form LEVs, DiI-labeled 4T1 cell membranes and DiO-labeled phospholipids were used in a FRET experiment, and a 1:1 weight ratio of cell membrane to liposome film was proved to be useful (Fig. 2G).

To explore the potential of LEVs in DOX and REV delivery, we examined the drug loading and stability of LEV@DOX@REV. As shown in Fig. S1, REV and DOX were incorporated into LEV@DOX@REV with a high loading efficiency of up to 58.2% ± 1.3% and 56.4% ± 2.6%, respectively. Furthermore, the in vitro release profile showed that LEV@DOX@REV released more drugs in acidic solution (pH 5.0; DOX release: 81.6% ± 4.7%; REV release: 80.6% ± 4.8%) compared with neutral solution (pH 7.0; DOX release: 24.8% ± 2.2%; REV release: 27.0% ± 3.2%), revealing that LEV@DOX@REV could effectively deliver DOX and REV to tumors, which are known to be acidic microenvironments (Fig. S2).

**Fig. 3.**
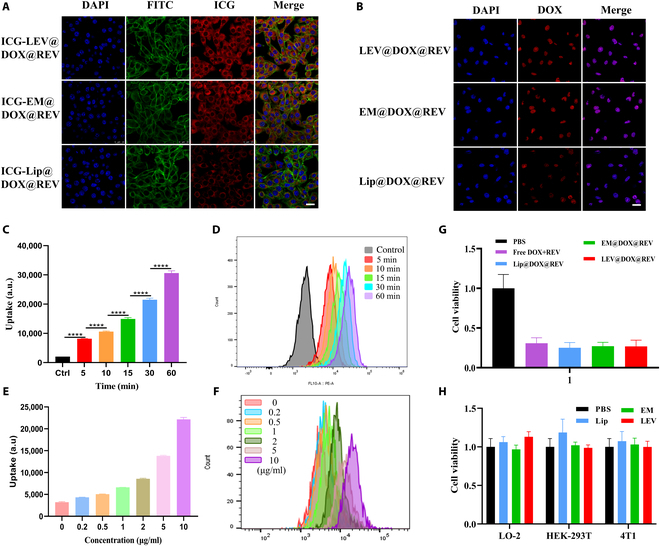
Confocal fluorescence images of tumor cells after incubation with ICG-labeled nanoparticles (A) or drug-loaded nanoparticles (B). Cell nuclei were stained blue with DAPI, and filamentous actin cytoskeletons were stained green with FITC phalloidin. Scale bar = 10 μm. Cellular uptake was determined by FCM at different time points (C and D) and different concentrations (E and F). (G) Viability of 4T1 cells following various treatments. (H) Viability of LO-2, HEK293T, and 4T1 cells incubated with PBS, EM, Lip, and LEVs.

To further validate its tumor-targeting ability, LEV@DOX@REV, along with Lip and EM, was incubated with 4T1 cells for 2 h. Then, confocal laser scanning microscopy (CLSM) was used to image the cells. The nanoparticles were labeled with ICG to trace their cell membrane distribution (Fig. 3A), or unlabeled fluorescent dye to monitor DOX signals to evaluate drug delivery (Fig. 3B). The results showed that both the LEV@DOX@REV group and the EM@DOX@REV group were highly fluorescent, while the Lip@DOX@REV group exhibited weak fluorescence, suggesting that cell membrane components contributed to cellular uptake. As the incubation time or drug concentration increased, tumor cell binding increased, suggesting that LEV@DOX@REV was ingested by cells in a time-dependent (Fig. 3C and D and S3) and dose-dependent (Fig. 3E and F) manner. We conducted a cell uptake experiment at a time point of 2 h after adding the drug, and a CCK-8 cytotoxicity experiment at 24 h. In fact, although the nanoparticles LEV and EM containing tumor cell membrane components significantly increased uptake rates in the early stages, liposomes were also able to achieve higher uptake rates in the final in vitro uptake experiment, as the in vitro uptake experiment was similar to the in vivo tumor in situ drug delivery strategy. As shown in Fig. S3, the uptake rates of ICG-LEV@DOX@REV and ICG-EM@DOX@REV gradually increased, peaking at 4 h; the uptake rate of ICG-Lip@DOX@REV also gradually increased, peaking at 8 h. As shown in Fig. S4, at 2 h of incubation, the cell viability of the LEV@DOX@REV group was 59.7% ± 2.5%, significantly higher than that of the Lip@DOX@REV group (1.45-fold); at 8 h of incubation, the cell viability of the LEV@DOX@REV group was 49.2% ± 5.8%, slightly higher than that of the Lip@DOX@REV group (1.15-fold).

As shown in Fig. 3G, the LEV@DOX@REV, Lip@DOX@REV, EM@DOX@REV, and free DOX+REV groups exhibited clear inhibition of cell viability compared with the PBS group. It is worth noting that there were no statistical differences in inhibitory activity between the 4 groups, indicating that the tumor-targeting nanoparticles offered little advantage in the in vitro experiments. As measured by the CCK-8 assay, LEVs exhibited negligible toxicity in all 3 cell lines, LO-2, HEK-293T, and 4T1 (Fig. 3H). The good safety profile confirmed that LEVs are a suitable nanocarrier for tumor therapy.

**Fig. 4.**
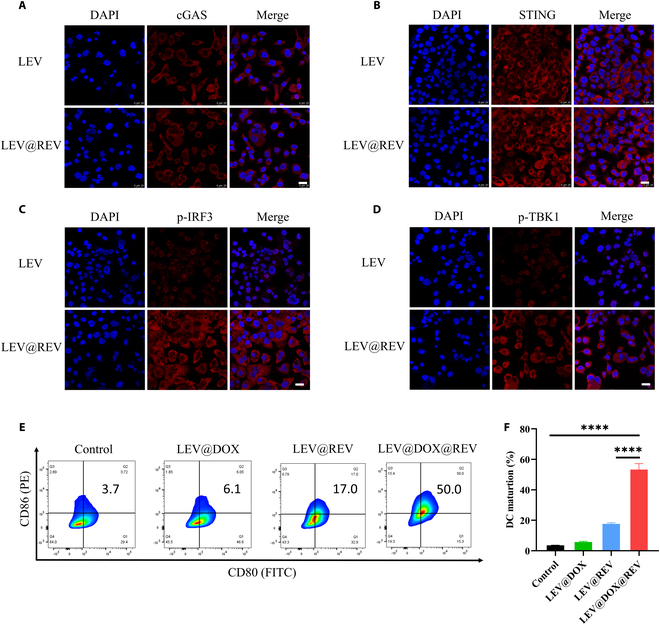
Representative images of PBS or LEV@REV-treated 4T1 cells stained with anti-cGAS (A), anti-STING (B), anti-IRF3 (C), and anti-TBK1 (D) antibodies. Scale bar = 50 μm. (E) Representative FCM plots and (F) quantification of DCs maturation after treatment with PBS, LEV@DOX, LEV@REV, or LEV@DOX@REV for 24 h.

**Fig. 5.**
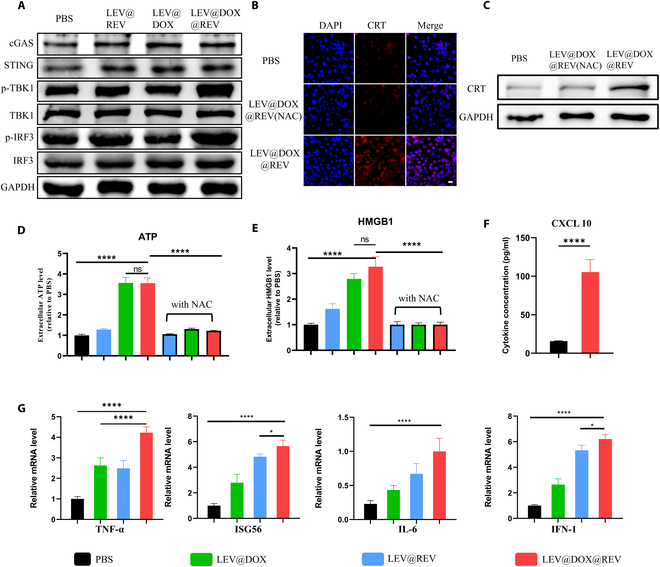
(A) WB analysis showing the impact of LEV@DOX@REV on the expression of the indicated proteins. (B) CRT release examined by CLSM (B) and WB (C). Scale bar = 50 μm. (D) The release of ATP from 4T1 cells with different treatments following incubation with or without NAC. (E) The secretion of HMBG1 in 4T1 cells treated with different treatments following incubation with or without NAC. (F) CXCL10 protein was measured by ELISA. (G) The expression of cGAS target genes, including those encoding TNF-α, ISG56, IL-6, and IFN-I, in 4T1 cells with the indicated treatment, was detected using real-time PCR.

To demonstrate that LEV@DOX@REV could effectively activate the cGAS/STING pathway and thus lead to the mobilization of a downstream signal cascade and transcriptional activities, we examined the protein expression of cGAS, STING, p-IRF3, and p-TBK1. Compared to the LEV group, the expression of cGAS and STING protein did not change significantly (Fig. 4A and B), but phosphorylated IRF3 (p-IRF3) and p-TBK1 protein was significantly increased after LEV@DOX@REV treatment (Fig. 4C and D), which is consistent with the results of WB (Fig. 5A). Given that LEV@DOX@REV exhibited great potential in cGAS/STING pathway activation and ICD induction, DC maturation was further assessed. DC maturation was significantly improved by LEV@DOX@REV compared with the control group as demonstrated by FCM (Fig. 4E and F). Notably, the LEV@DOX@REV group (~50.0% DC maturation) was able to cause remarkably increased DC maturation compared with the other treatments (LEV@DOX group: ~6.1% DC maturation; LEV@REV group: ~17.0% DC maturation), implying that antitumor immunotherapy based on LEV@DOX@REV triggered robust cytotoxic T lymphocyte (CTs) responses.

To verify the activation of the cGAS/STING signaling pathway, the protein expression levels of downstream markers, such as IRF3, p-IRF3, TBK1, and p-TBK1, were determined by WB. It was found that p-IRF3 and p-TBK1 were augmented in the LEV@DOX@REV groups, confirming that LEV@DOX@REV could cause much stronger cGAS/STING pathway activation (Fig. 5A). The CRT signal, a marker for ICD, stimulates DCs maturation and immune activation by signaling “eat me”. LEV@DOX@REV treatment was associated with the increased expression of CRT on cell membranes compared with the control group. LEV@DOX@REV was shown to significantly enhance CRT exposure on the cell membrane by CLSM (Fig. 5B) and WB (Fig. 5C). In comparison with PBS, LEV@DOX@REV significantly increased the secretion of ATP into the extracellular milieu of cancer cells (*P* < 0.05, Fig. 5D), and there was a significant difference between LEV@DOX@REV and PBS regarding HMGB1 release from the nucleus into the extracellular milieu (Fig. 5E). Reactive oxygen species (ROS) can elicit immunogenicity associated with ICD [25], and ROS-inducing strategies may enhance the efficacy of ICD [26–28]. A significant reduction in ICD hallmark activity was observed after LEV@DOX@REV treatment of cancer cells pretreated with NAC, demonstrating that ROS production may be critical for LEV@DOX@REV ICD induction, likely because DOX induces ICD through endoplasmic reticulum stress as well as ROS production [29]. NAC is a cysteine prodrug widely used as an antioxidant in cellular, animal, and clinical research. It is generally believed that NAC is an acetylation precursor of GSH, which can be directly reacted with ROS through thiol groups or converted into sulfur species that can effectively clear ROS under the action of related enzymes.

Furthermore, LEV@DOX@REV significantly up-regulated the CXCL10 mRNA level compared with the control group, which is a critical chemokine of antitumor T cell activation and recruitment (Fig. 5F). A PCR assay was carried out to determine the effect of LEV@DOX@REV on cGAS/STING signal transduction in 4T1 cells exposed to LEV@DOX@REV. The results showed that LEV@DOX@REV significantly increased the secretion of TNF-α, ISG56, IL-6, and IFN-I (Fig. 5G).

**Fig. 6.**
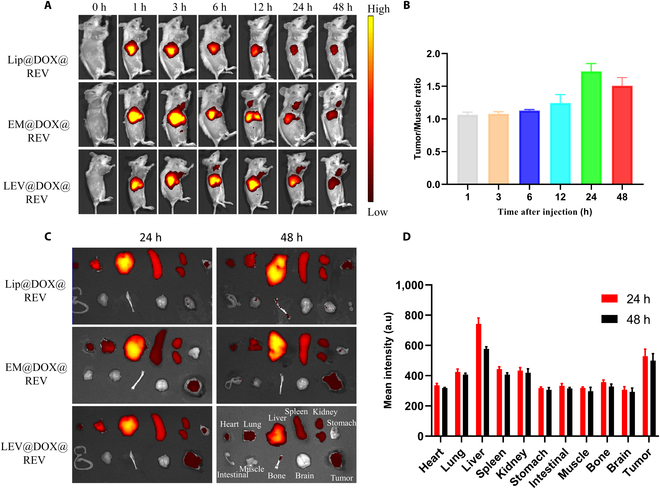
Biodistribution and antitumor efficacy of LEV@DOX@REV in 4T1 tumor-xenograft mice. (A) In vivo NIR fluorescent images of tumor-bearing mice after injection of ICG-labeled nanoparticles. (B) Ex vivo NIR fluorescent images of the main organs and tumors at 24 and 48 h post-injection. (C) The tumor/muscle ratio of the LEV@DOX@REV group at different time points. (D) Quantitative biodistribution of LEV@DOX@REV in the main organs at 24 and 48 h post-injection.

LEV@DOX@REV biodistribution in mice was assessed using in vivo fluorescence imaging (Fig. 6A). Fluorescence was observed at the tumor location within 3 h of injection, and its intensity increased over time, peaking at 24 h when the tumor to muscle (T/M) ratio was at its highest (1.8 ± 0.2; Fig. 6B). After 48 h, a significant fluorescent signal was still visible in the tumor, demonstrating long-term persistence of the biomimetic LEVs. Further confirmation of nanoparticle accumulation was provided by fluorescence images obtained ex vivo (Fig. 6C and D). Notably, the liver also showed strong fluorescence, likely due to the clearance by the liver of LEV@DOX@REV nanoparticles.

**Fig. 7.**
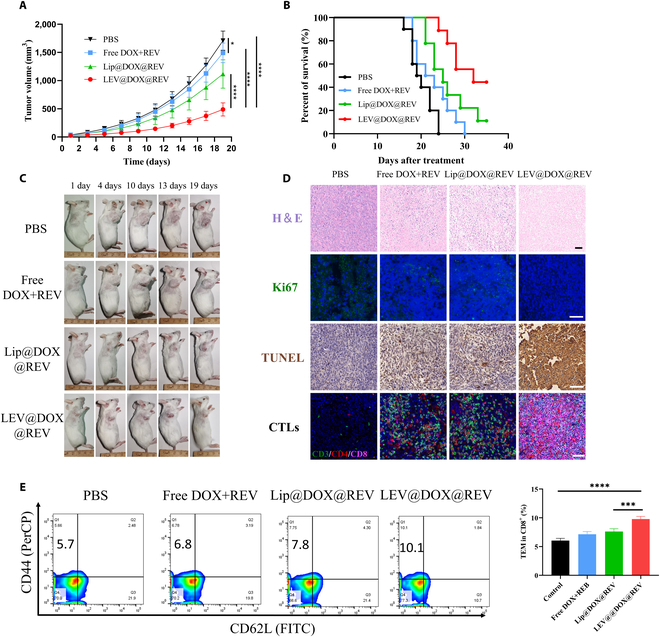
In vivo therapeutic properties of LEV@DOX@REV. The detection of tumor volume (A, *n* = 8) and survival curves (B, *n* = 10) after the indicated treatments. (C) Photographs of the tumors extracted from mice after the indicated treatments. (D) H&E, Ki67, TUNEL, and CTL staining of tumors. Scale bar = 50 μm. Representative images (E) showing memory T cells (CD8^+^CD44^+^CD62L^˗^) in splenocytes, as measured by FCM and quantitative analysis (*n* = 4).

For 19 days after systemic treatment, tumor volume was quantified every 2 days for all groups to assess their anti-tumor activities *in vivo*. There were 4 groups of mice bearing tumors (*n* = 8): (a) PBS; (b) Free DOX+REV; (c) Lip@DOX@REV; and (d) LEV@DOX@REV. LEV@DOX@REV significantly inhibited tumor growth compared with the other groups, as shown in Fig. 7A. At 19 days post-treatment, the mean tumor volume in the LEV@DOX@REV group (488.8 ± 177.7 mm^3^) was significantly smaller than that in the Lip@DOX@REV group (1,111.8 ± 251.9 mm^3^), which indicated that the cell membrane components contained in the nanoparticles effectively increase the tumor targeting ability (Fig. 7A), and this result was concordant with the *in vivo* imaging results (Fig. 6A). Moreover, the LEV@DOX@REV group survived for 35 days compared with 24 days for the PBS group (Fig. 7B), and LEV@DOX@REV (55.6%) improved survival significantly compared with Lip@DOX@REV (11.1%). Photographs of the tumors extracted from mice after the indicated treatments are shown in Fig. 7C. Furthermore, treatment with LEV@DOX@REV boosted the innate and adaptive antitumor immune responses by strongly activating the cGAS/STING pathway, and the expression levels of related proteins were higher than those in the Lip@DOX@REV group (Fig. S5). Notably, no statistical difference was found between the LEV@DOX@REV group and the EM@DOX@REV group, indicating that the design of this hybrid nano would be expected to confer the advantages of excellent safety, easy preparation, and tumor-targeting ability without reducing the therapeutic effect. As shown by H&E staining, both LEV@DOX@REV and EM@DOX@REV induced the same high rate of apoptosis (Fig. S6).

Next, hematoxylin and eosin (H&E) staining, terminal deoxynucleotidyl transferase dUTP nick end labeling (TUNEL) staining, and Ki-67 immunohistochemical staining were performed (Fig. 7D). LEV@DOX@REV treatment caused the most severe damage and necrosis compared with Free DOX+REV and Lip@DOX@REV. Additionally, we detected the level of antitumor immune cells infiltrating tumors and found that CD8+ T cells significantly infiltrated tumors in the LEV@DOX@REV group, indicating that the adaptive immune system was activated. Given the crucial role of immune memory in long-term antitumor benefits, we next assessed the immune memory generated by LEV@DOX@REV (Fig. 7E). We assayed the populations of immune memory cells in splenocytes after treatment. Compared with the control group, the percentages of effector memory T cells (CD8^+^CD44^+^CD62L^−^) in the LEV@DOX@REV group showed a significant increase. These results indicate that LEV@DOX@REV can generate antigen-specific immune memory, which provides a long-term tumor prevention effect.

**Fig. 8.**
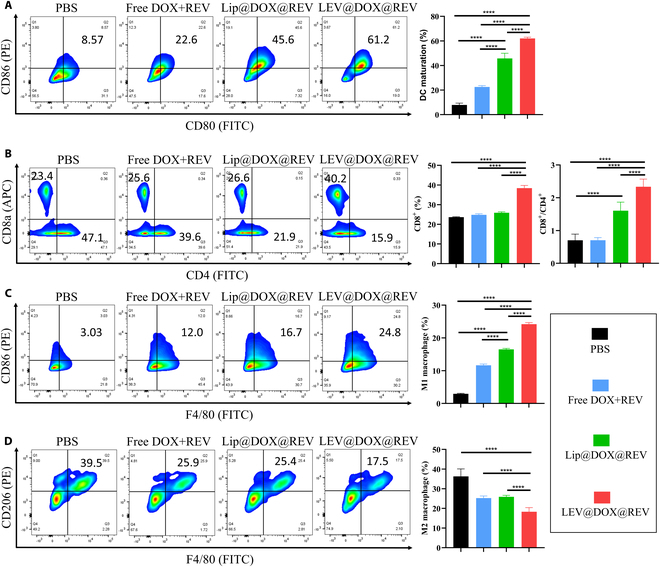
Immune responses to LEV@DOX@REV-mediated therapy in the tumor-bearing mouse model. Representative FCM plots and quantification analysis of CD80^+^CD86^+^ DCs in the spleen (A), CD3^+^CD8^+^ or CD3^+^CD4^+^ T cells (B), F4/80^+^CD86^+^ macrophages (C), and F4/80^+^CD206^+^ macrophages (D) in 4T1 tumors after different treatments (*n* = 4).

To further understand the anti-tumor immune mechanisms triggered by LEV@DOX@REV, FCM was used to analyze the percentages of various immunocytes in tumors. We first investigated the number of mature DCs in the spleen after different treatments (Fig. 8A). The proportion of mature DCs in the LEV@DOX@REV group (62.0% ± 1.0%) was greater than that in the control group (7.9% ± 1.5%), verifying that LEV@DOX@REV could induce DC maturation in vivo. It is well known that mature DCs act as a bridge between tumor antigens and T lymphocyte responses, which facilitates the infiltration of CTLs within the tumor. Consistent with this, LEV@DOX@REV induced 38.4% ± 1.3% CD8+ T cell infiltration into tumors, which was 1.6 times higher than that observed in the control group (Fig. 8B). Furthermore, we investigated the potential of LEV@DOX@REV in re-educating tumor-associated macrophages into M1-like phenotypes. LEV@DOX@REV greatly increased the percentage of M1-like tumor-associated macrophages and decreased the percentage of M2-like tumor-associated macrophages in tumors (Fig. 8C and D).

**Fig. 9.**
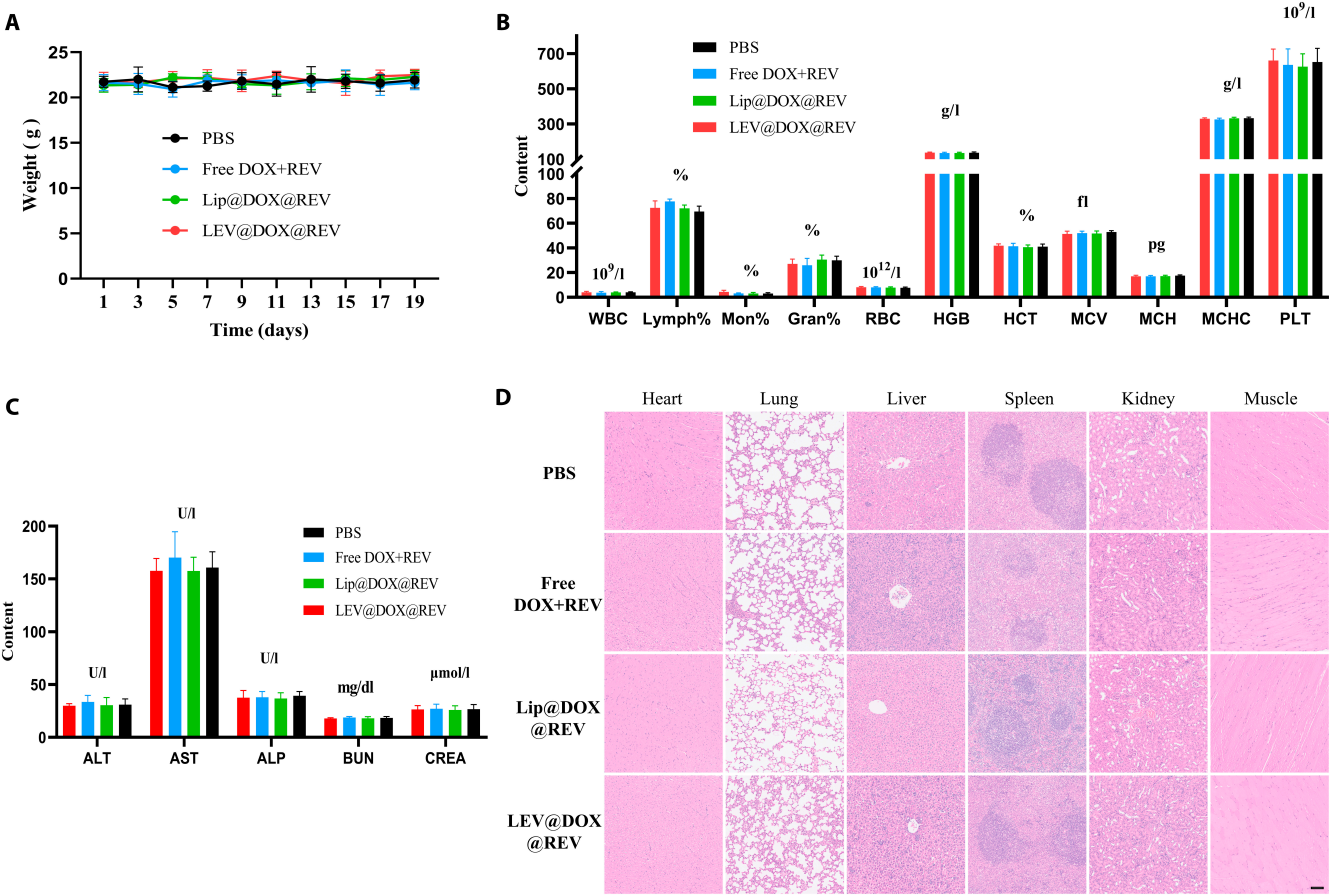
Biosafety evaluation. (A) Changes in the body weight of mice at 19 days. (B) Blood routine parameter data. (C) Blood biochemistry data including liver function markers ALT, AST, and ALP and kidney function markers BUN and CRE. (D) H&E-stained slice images of major organs. Scale bar = 100 μm.

During the 19-day experiment, none of the groups of mice showed detectable weight loss (Fig. 9A), and neither routine blood parameters nor blood biochemistry parameters showed significant variations outside of the normal ranges (Fig. 9B and C). There were no signs of histological damage, as determined by representative H&E staining (Fig. 9D). Based on these results, LEV@DOX@REV was highly biocompatible.

**Fig. 10.**
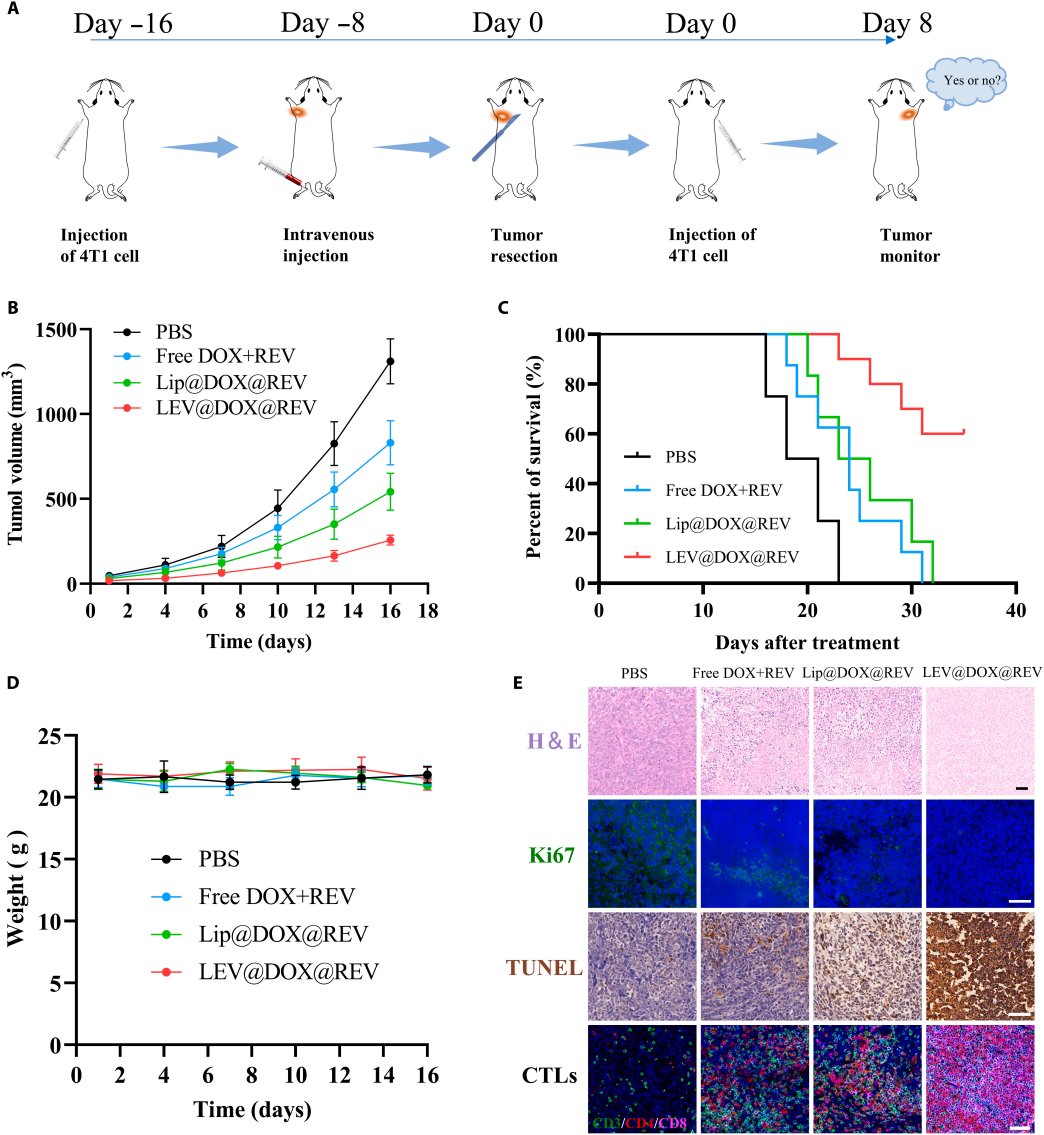
Distant tumor growth inhibition effect of LEV@DOX@REV. (A) Schematic of the administration protocol. The detection of tumor volume (B, *n* = 8), survival curves (C, *n* = 10), and weight changes (D, *n* = 5) after the indicated treatments. (E) H&E, Ki67, TUNEL, and CTL staining of tumors. Scale bar = 50 μm.

Successful tumor therapy requires the participation of the adaptive immune response to prevent secondary tumors, which leads to the long-term eradication of resistant and recurrent cancer. We inoculated the secondary 4T1 tumors after therapy into the distal side of the mice. Compared with mice treated with the free DOX+REV group, mice treated with LEV@DOX@REV exhibited a remarkable inhibition of secondary tumor growth (Fig. 10A and B). Besides, LEV@DOX@REV treatment significantly prolonged survival of tumor-bearing mice (Fig. 10C). The body weight of the mice did not change significantly during treatment (Fig. 10D). LEV@DOX@REV-treated mice had a high number of CD8^+^ T cells infiltrating the secondary tumor region compared with mice in the other groups, indicating tumor-specific immunity (Fig. 10E). A large area of apoptosis and necrosis was observed in the secondary tumors of the LEV@DOX@REV-treated group, which demonstrated the ability of CD8^+^ T cells to kill tumor cells. To summarize, LEV@DOX@REV facilitated systemic immune responses that prevented tumor recurrence and metastasis by activating the cGAS/STING pathway.

**Fig. 11.**
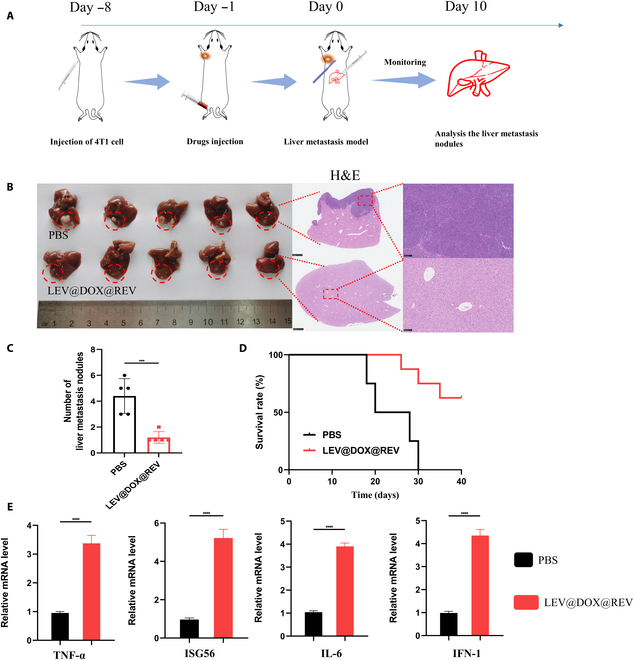
In vivo therapeutic efficacy against 4T1 liver metastasis. (A) Illustration of the treatment schedule for evaluating the anti-metastasis effect. (B) Photographs of excised livers and H&E staining. Scale bar, left: 1 mm, right: 100 μm. (C) Number of liver metastasis nodules after different treatments (*n* = 5). (D) Survival curves of mice after different treatments during a 40-day observation period (*n* = 10). (E) Cytokine levels of TNF-α, ISG56, IL-6, and IFN-I in the serum of mice (*n* = 4).

To test whether LEV@DOX@REV treatment could also inhibit tumor metastasis in vivo, we developed a 4T1 liver metastasis model. The drug administration scheme is described in Fig. 11A. On day 10, mice were euthanized to analyze their liver metastases. As the morphology and H&E staining of collected livers indicated (Fig. 11B), the control group had a mass of liver metastasis nodules, some reaching 0.7 cm in diameter. Significantly, there were only 1 to 2 metastasis nodules in the LEV@DOX@REV group compared with 3 to 6 nodules in the control group (Fig. 11C). In the tumor-bearing mice, a 60% survival rate was seen in the LEV@DOX@REV group after 40 days, whereas none of the mice in the control groups survived (Fig. 11D). Furthermore, we investigated the serum levels of various immune cytokines following different treatments. Notably, after LEV@DOX@REV treatment, the levels of TNF-α, ISG56, IL-6, and IFN-I in serum reached their highest values (Fig. 11E). Taken together, the excellent systemic immune responses triggered by LEV@DOX@REV offer a favorable strategy for effective 4T1 liver metastasis inhibition.

## Discussion

The application of cell membrane-coated nanoparticles in targeting strategies has gained increasing attention recently. Tumor targeting, enhanced biocompatibility, and effective drug loading can be achieved by different tumor cell membranes [30,31]. Extrusion technology is commonly used to homogenize the size distribution of the cell membrane-coated nanoparticles. Nanovesicles with exosome-like properties have been widely developed using this strategy in recent years [32]. Nanovesicles with similar physical properties and biological characteristics as natural exosomes could be produced by crushing cultured cells through polycarbonate membrane filters of diminishing pore sizes (0.5, 0.2, and 0.1 μm) [33,34]. Several properties of nanovesicles are similar to those of natural exosomes, including the physical attributes, key protein markers, and therapeutic potential [32]. The most fascinating feature of the extrusion approach is that the production yield of nanovesicles is considerably higher than that of exosomes purified from the same number of cells (roughly 100 times higher) [33]. It is still challenging for conventional nanoparticles to be absorbed into target tissues, since they are cleared quickly by blood circulation, easily recognized by the immune system, and accumulate at low concentrations in the target tissues [21]. Proteins present on the surface of cell membranes have led to cell membrane coating being recognized as a viable means to overcome these limitations [21,31]. Biomimetic nanoparticles with membrane coatings are used to create therapeutic devices with a nanoparticle core covered with a membrane generated from a variety of cell types [35]. Biomimetic nanoparticles have attracted a lot of attention in recent years due to their properties as customized nanomaterials and smart nanoparticles, with potential applications in cancer therapy [36]. In this study, we designed cell membrane-coated nanoparticles loaded with DOX and REV combined immunotherapy and ICD therapy. Because of their nanoscale size, LEV@DOX@REV preferentially accumulate in tumors via the EPR effect [37], and as a result of surface proteins, LEV@DOX@REV possesses the unique property of homologous binding [38,39]. Our data showed that LEV@DOX@REV offers the advantages of excellent stability and strong tumor targeting ability. In vitro and in vivo experiments confirmed that LEV@DOX@REV could activate the cGAS/STING signal pathway and ICD, activate immune cells, and release cytokines, thus leading to tumor apoptosis through chemotherapy combined with immunotherapy. Numerous studies have shown that immunotherapy combined with chemotherapy has promising prospects for cancer therapy [40].

Nanoparticles formed from different cell membranes typically have some unique properties [41]. In this study, we used cancer cells to prepare drug-loaded nanoparticles (LEV@DOX@REV), which have been widely investigated because of their tumor-targeting properties [38,42].

Cancer cells often have a mutual affinity for other cancer cells, which is thought to contribute to tumor development and metastasis [43]. This homotypic binding allows nanoparticles to be developed as excellent drug carriers for delivering therapeutics directly to tumors [44]. To reduce the potential side effects induced by tumor cell membrane components, we employed cell membrane extraction technology to prepare the nanoparticles LEV@DOX@REV, and a 1:1 weight ratio of cell membrane to liposome film was proven to be useful for LEVs by a FRET study. Our data indicated the excellent stability and tumor-targeting properties of LEV@DOX@REV (Fig. 2).

Induction of ICD could also be mediated by ROS or DOX. It has been demonstrated by Deng et al. [45] that the PDT effect is capable of triggering ICD and enhancing cancer immunotherapy. To improve tumor immunogenicity, ICD is a proven strategy [46]. ICD occurs when specific inducers harness the host immune system to recognize and kill cancer cells by causing endoplasmic reticulum stress [47]. The damage-associated molecular patterns (DAMPs) are released by dying tumor cells, which primarily consist of CRT, HMGB1, and ATP [48]. Long-term protection against tumor relapse and metastasis can be obtained following the induction of an adaptive immune response. As a result of the immune response induced following treatment with LEV@DOX@REV, tumor recurrence was prevented over the long term. To detect CD8^+^ T cells in secondary tumors, immunofluorescence staining was performed. A large area of apoptosis and necrosis was observed in the secondary tumors of the LEV@DOX@REV-treated group, which demonstrated the ability of CD8^+^ T cells to kill tumor cells (Fig. 10E).

To summarize, LEV@DOX@REV facilitated systemic immune responses that prevented tumor recurrence and metastasis by activating the cGAS/STING pathway. Furthermore, by triggering “eat me” signals in tumor cells, DOX might help DCs phagocytize apoptotic tumor cells [49]. Moreover, the synergistic effects of DOX and REV may enhance tumor-specific adaptive immunity by triggering DNA damage, activating cGAS/STING, and assisting DCs in absorbing tumor-associated antigens from dying tumor cells.

As a final step, biosafety was systematically tested. No significant differences in body weight were detected among mice in the different groups (Fig. 9A). All blood analysis parameters were within normal ranges, indicating that treatment did not cause inflammation (Fig. 9B). Liver function markers, kidney function markers, and other blood biochemical indicators were all within reference ranges (Fig. 9C). In all groups, H&E staining of major organs revealed no distinct morphological differences or damage (Fig. 9D). We therefore conclude that LEV@DOX@REV nanovesicles possess high biosafety and potential applications in cancer therapy.

However, limitations of this study should also be sincerely pointed out. FRET, as a “spectroscopic ruler”, is most sensitive to changes in the distance between the donor and acceptor fluorophores. As the distance increases, FRET shows significant weakening. For subsequent studies, FRET could be performed with the donor and acceptor fluorophore-labeled components in LEVs to obtain the optimized ratio. Besides, the loading amount of DOX or REV should be optimized. Thus, the preparation and drug loading methods for the nanomaterials will be optimized in our subsequent research. LEV@DOX@REV still achieved excellent antitumor effects even with only satisfactory drug loading.

In this study, we prepared nanocomposite LEV@DOX@REV by fusion of cell membranes, phospholipids, DOX, and REV, to realize tumor-targeting delivery and chemo-immunotherapy. The novel hybrid lipid-cell membrane nanovesicles offer the advantages of excellent safety, easy preparation, and tumor-targeting ability, making them ideal therapeutic agents for cancer. Through the EPR and “homing” targeting effect, LEV@DOX@REV nanovesicles were found to effectively locate and accumulate at the tumor site after intravenous injection. By triggering “eat me” signals in tumor cells, DOX might help DCs phagocytize apoptotic tumor cells, while REV can activate the cGAS/STING pathway, increase CTL infiltration, and enhance the effect of chemotherapy and immunotherapy. As a result of activating the cGAS/STING pathway, LEV@DOX@REV could prevent tumor relapse and metastasis. Finally, the results of routine blood indexes, serum biochemical indicators, and H&E staining of the main organs confirmed that LEV@DOX@REV offer excellent safety. Our study lays the foundation for the development of chemo-immunotherapy to treat breast tumors.

## Data Availability

The analyzed datasets generated during this study are available from the corresponding authors on reasonable request.
