# Supplementary material for: Cell Membrane Hybrid Lipid Nanovesicles Enhance Innate Immunity for Synergistic Immunotherapy by Promoting Immunogenic Cell Death and cGAS Activation
Source: Biomater Res. 2024 Jul 2;28:0038. doi: 10.34133/bmr.0038 (PMC11168305; doi:10.34133/bmr.0038)
Supplement: Supplementary 1 — Figs. S1 to S6 Tables S1 and S2 [file bmr.0038.f1.zip › supporting information.docx]

Table S1 Antibodies used in the study

| Antibodies | Source | Catalog |
| --- | --- | --- |
| Anti-mouse cGAS | Abclonal | A8335 |
| Anti-mouse STING | Abclonal | A21051 |
| Anti-mouse p-TBK1 | Abclonal | AP0847 |
| Anti-mouse TBK1 | Abclonal | A3458 |
| Anti-mouse p-IRF3 | Abclonal | AP0857 |
| Anti-mouse IRF3 | Abclonal | A2172 |
| Anti-mouse CRT | Abclonal | A1066 |
| Anti-mouse CD44 | Abclonal | A1351 |
| Anti-mouse Histone H3 | Abclonal | A2348 |
| Anti-mouse β-Tubulin | Abclonal | AC008 |
| Anti-mouse GAPDH | Abclonal | AC001 |
| ABflo® 594-conjugated Goat anti-Mouse IgG (H+L) | Abclonal | AS054 |
| PE Anti-Mouse CD206 | Elabscience | E-AB-F1135D |
| PE Anti-Mouse CD86 | Elabscience | E-AB-F0994UD |
| FITC Anti-Mouse CD80 | Elabscience | E-AB-F0992UC |
| FITC Anti-Mouse F4/80 | Elabscience | E-AB-F0995UC |
| PE anti-mouse CD3 | BioLegend | 100205 |
| FITC anti-mouse CD4 | BioLegend | 100405 |
| APC anti-mouse CD8a | BioLegend | 100711 |
| PerCP Anti-Mouse CD44 | BioLegend | 103035 |
| FITC Anti-Mouse CD62L | BioLegend | 104405 |

Table S2 Primers used for RT-qPCR

| Gene name | | Sequence (5’-3’) |
| --- | --- | --- |
| *IL-6* | Forward primer | GAGGATACCACTCCCAACAGACC |
|  | Reverse primer | AAGTGCATCATCGTTGTTCATACA |
| *TNF-α* | Forward primer | AAGCCTGTAGCCCACGTCGTA |
|  | Reverse primer | GGCACCACTAGTTGGTTGTCTTTG |
| *Isg56* | Forward primer | ACAGCAACCATGGGAGAGAATGCTG |
|  | Reverse primer | ACGTAGGCCAGGAGGTTGTGCAT |
| *IFN-1* | Forward primer | TCCTGCTGTGCTTCTCCACCACA |
|  | Reverse primer | AAGTCCGCCCTGTAGGTGAGGTT |


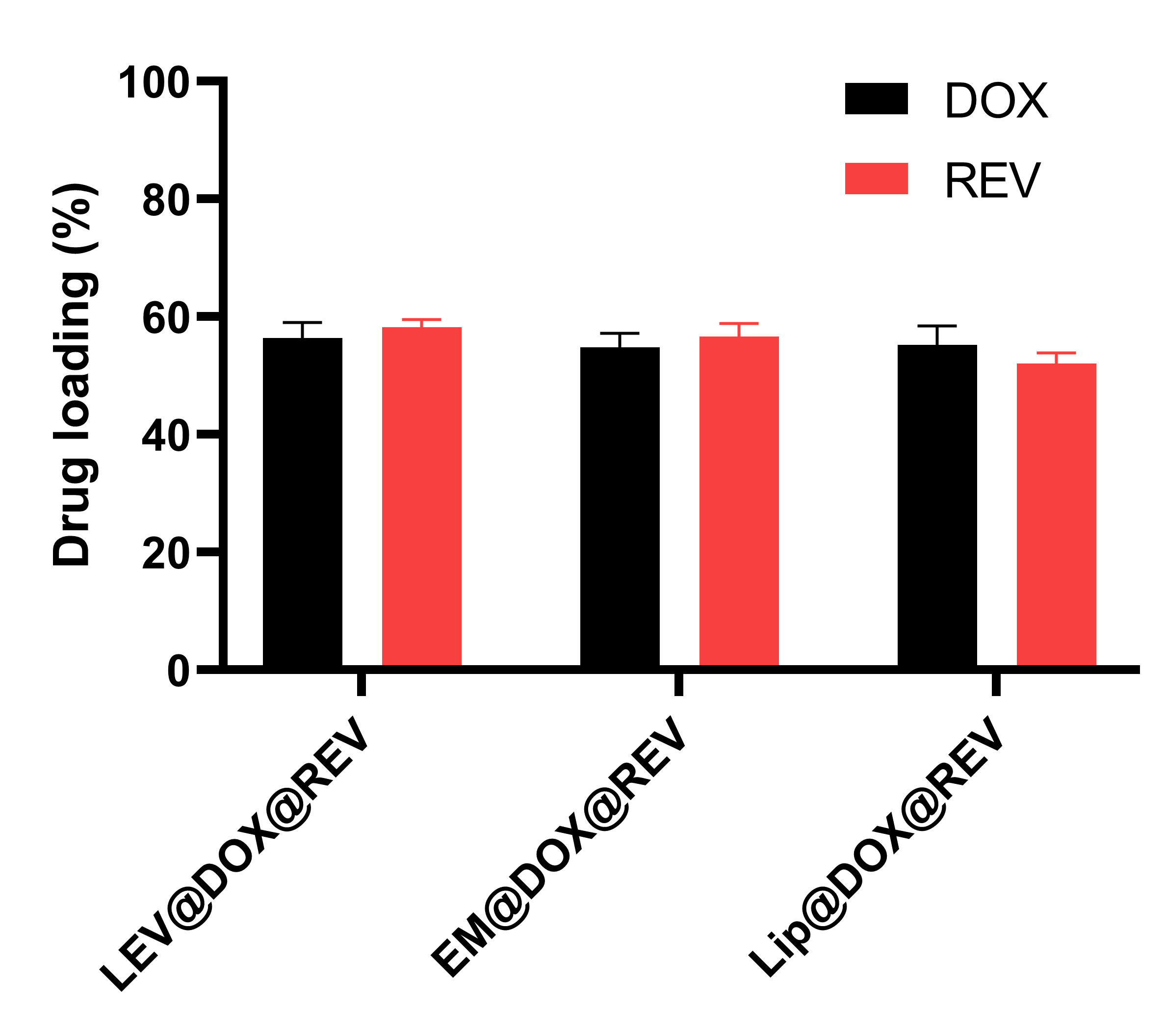


Fig. S1 DOX and REV loading of LEV@DOX@REV, EM@DOX@REV and Lip@DOX@REV (n = 4).


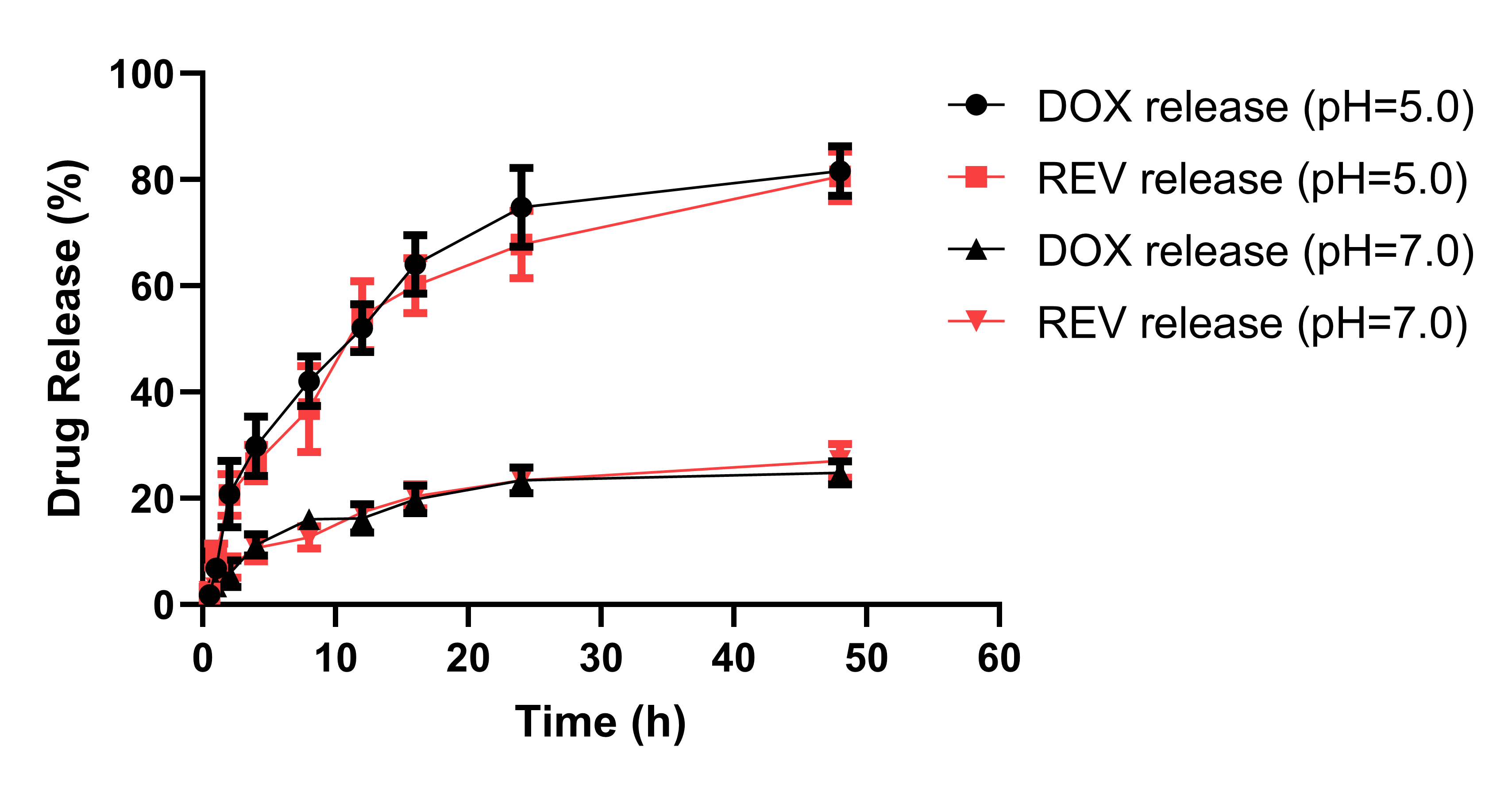


Fig. S2 DOX release profile and REV release profile from LEV@DOX@REV in different conditions (n = 4).


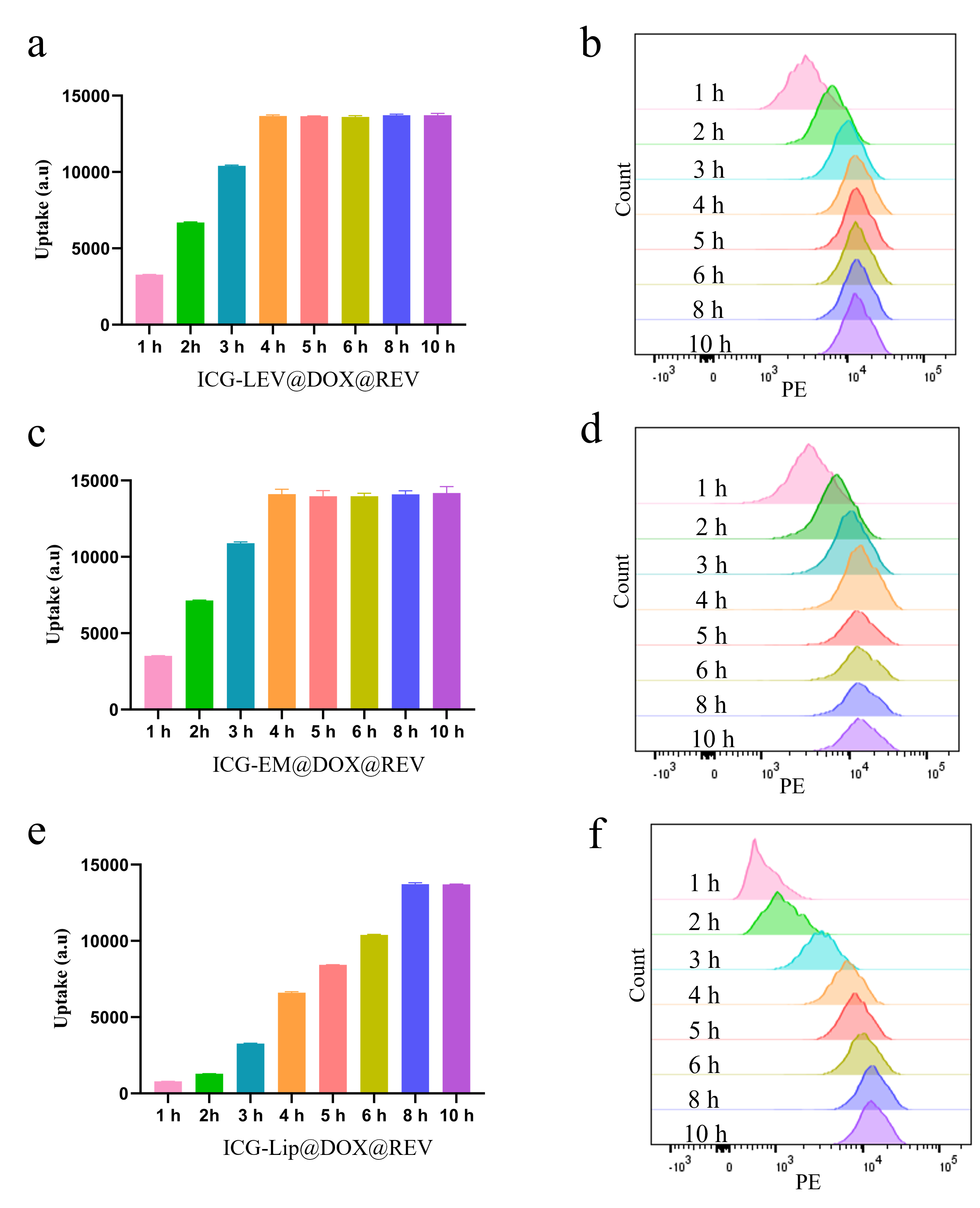


Fig. S3 Cellular uptake was determined and flowcytometry at different time points.

Representative FCM and quantification analysis of cellular uptake in ICG-LEV@DOX@REV (a, b), ICG-EM@DOX@REV (c, d), and ICG-Lip@DOX@REV (e, f).





Fig. S4 Viability of 4T1 cells incubated with LEV@DOX@REV or Lip@DOX@REV for 2 hours or 8 hours.


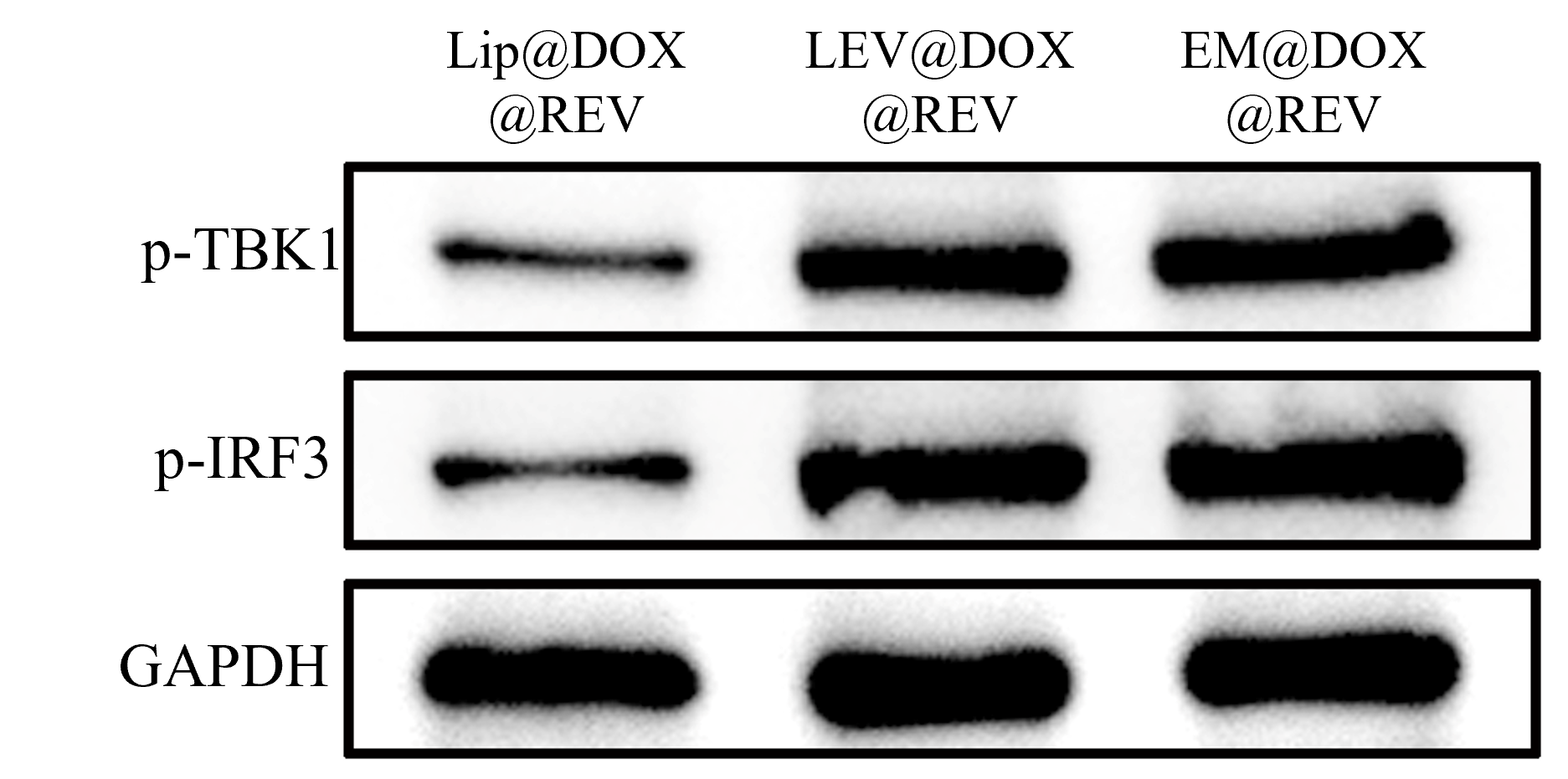


Fig. S5 WB analysis showing the impact of Lip@DOX@REV, LEV@DOX@REV, and EM@DOX@REV on the expression of the indicated proteins in tumors.


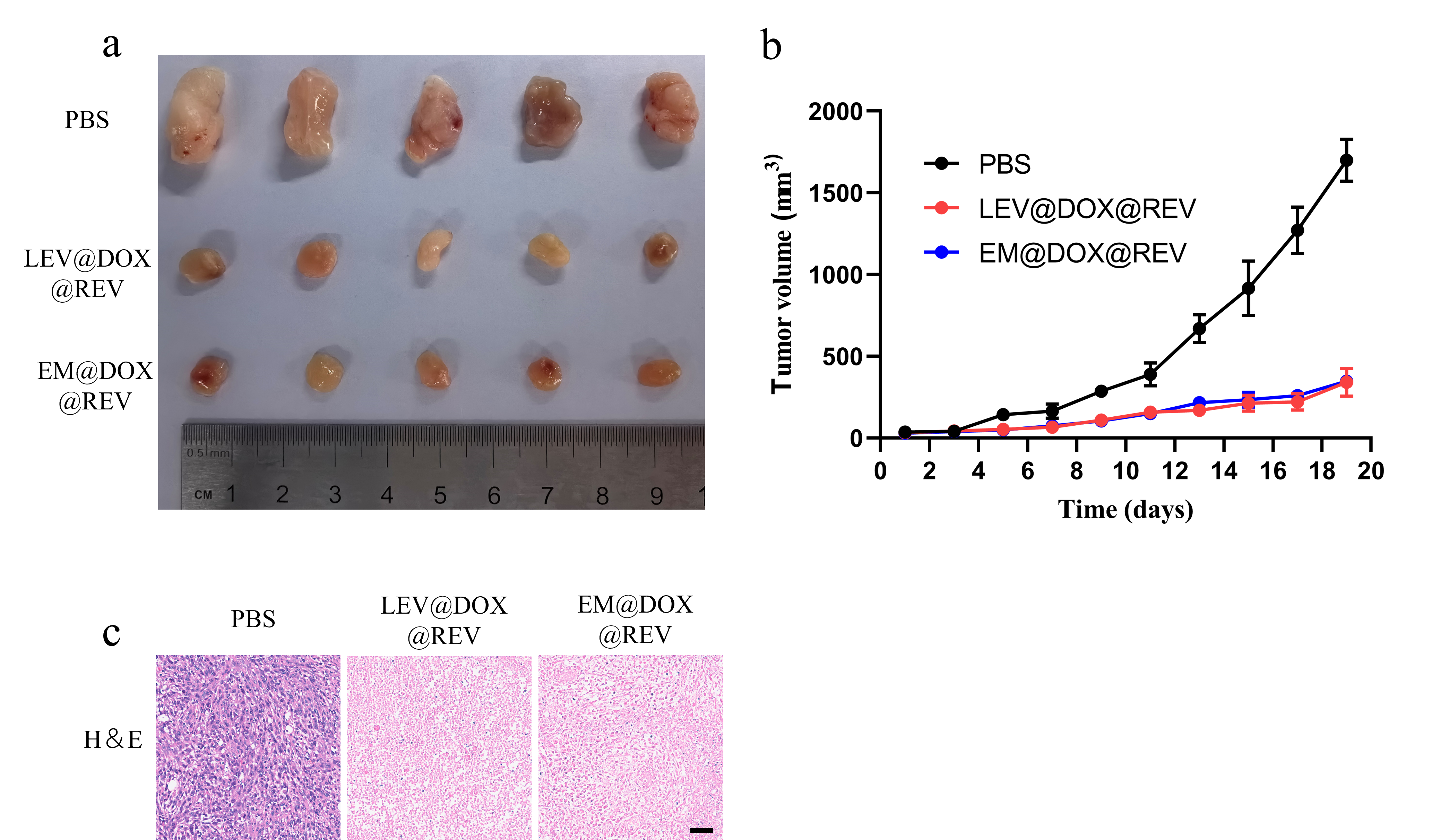


Fig. S6 (a) Representative tumor photographs of various treatments. (b) The detection of tumor volume after the indicated treatments (n = 5). (c) H&E staining of tumors. Scale bar: 50 μm.

**Materials**The following cell lines were used: murine 4T1 mammary carcinoma cell, normal liver cell line L-O2, human embryonic kidney 293T cells. Cells were cultured in RPMI-1640 medium or DMEM medium supplemented with 10% FBS and 1% penicillin/streptomycin at 37℃ with 5% CO_2_.

RPMI-1640 medium, DMEM medium, trypsin-EDTA, phosphate buffer (PBS), fetal bovine serum (FBS), dimethyl-sulfoxide (DMSO), 100 U/mL penicillin and 100 mg/mL streptomycin were obtained from SolarBio (Beijing, China). Reversine and Doxorubicin was purchased from Macklin Inc. (Shanghai, China). Tris-Glycine SDS Buffer (pH8.3, 10×) Tris-Glycine Transfer Buffer (pH8.3, 10×), TBST (pH8.0, 10×) and TRIzon Reagent for RNA extraction were obtained from CW Biotech (Beijing, China). Collagenase IV, hyaluronidase, DNase I,4’,6-diamidino-2-phenylindole (DAPI), monoclonal fluorescein isothiocyanate (FITC)-labeled phalloidin, RIPA lysis buffer and pheylmethylsulfonyl fluoride (PMSF) were purchased from SolarBio (Beijing, China). CCK8 kit, BCA kit and SDS–PAGE gel preparation kits were purchased from Beyotime Biotechnology (Shanghai, China). All ELISA kits were purchased from Multi Sciences (Lianke) Biotech Co., Ltd (Hangzhou, China). ECL Plus chemiluminescence assay kit was purchased from UElandy (Suzhou, China).4% paraformaldehyde fixative solution was purchased from (Servicebio, Wuhan, China).
