# Supplementary figures and images for: Cell Membrane Hybrid Lipid Nanovesicles Enhance Innate Immunity for Synergistic Immunotherapy by Promoting Immunogenic Cell Death and cGAS Activation
Source: Biomater Res. 2024 Jul 2;28:0038. doi: 10.34133/bmr.0038 (PMC11168305; doi:10.34133/bmr.0038)

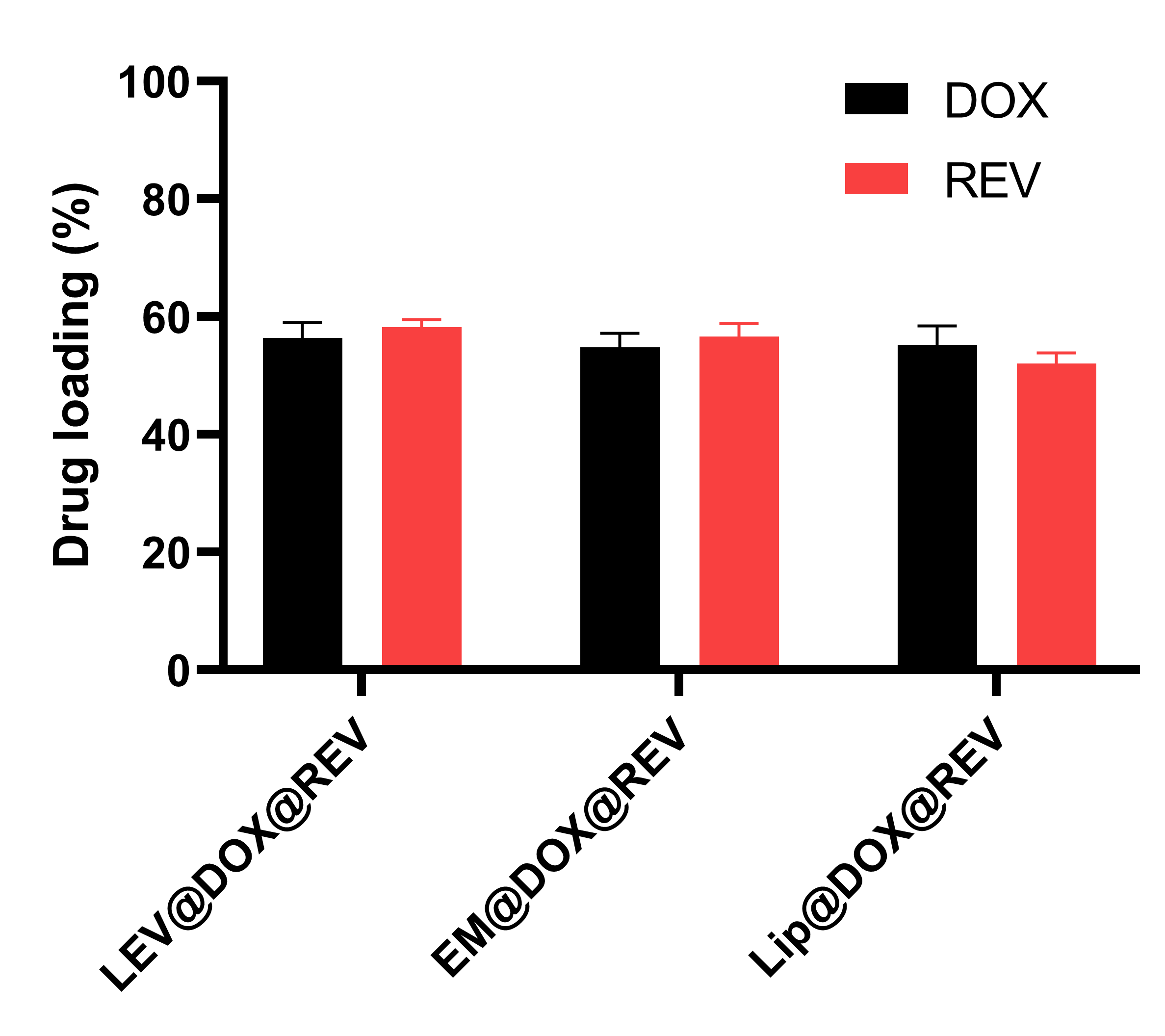

Supplement: Supplementary 1 — Figs. S1 to S6 Tables S1 and S2 [file bmr.0038.f1.zip › Fig. S1.tif]

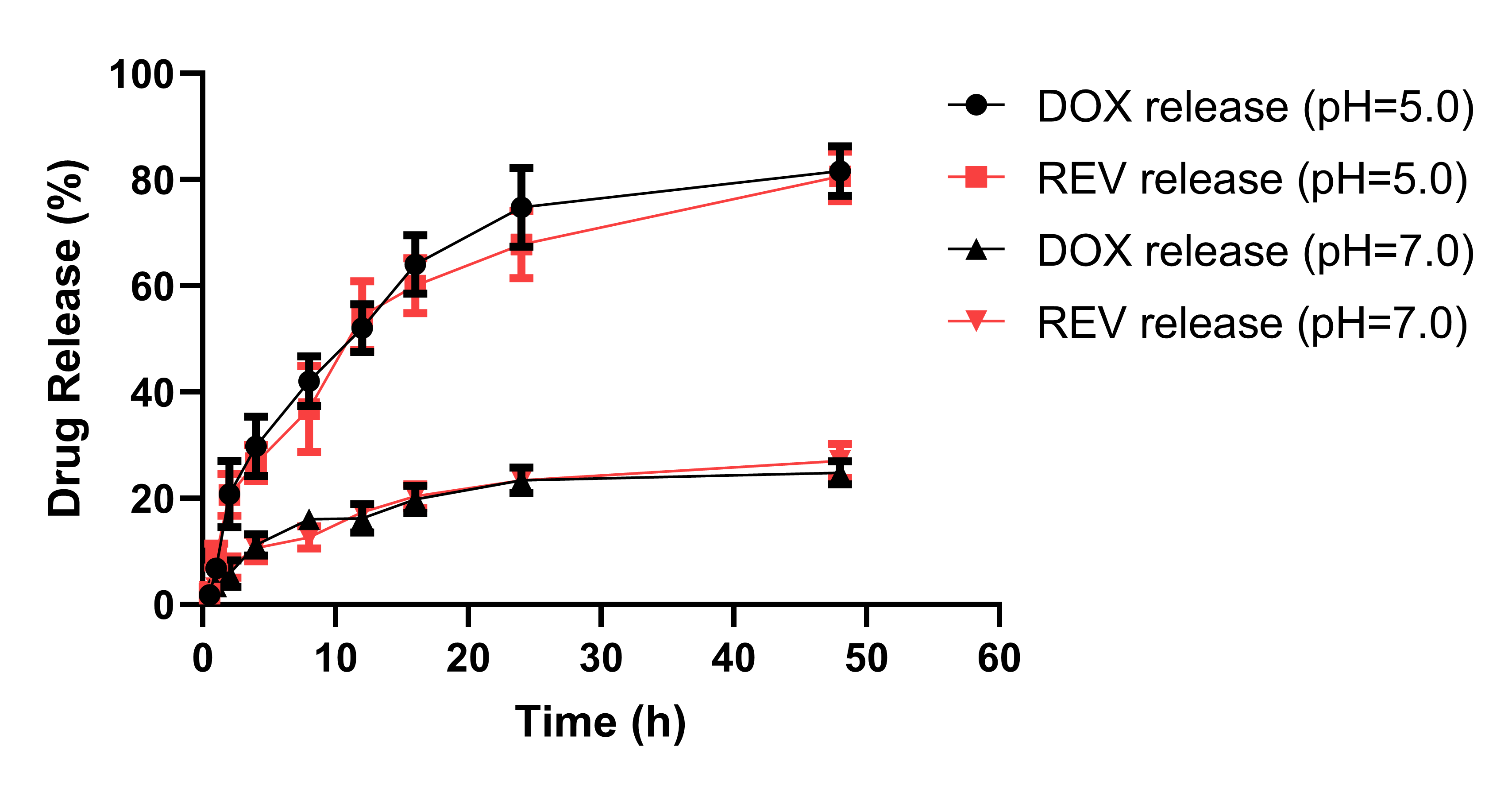

Supplement: Supplementary 1 — Figs. S1 to S6 Tables S1 and S2 [file bmr.0038.f1.zip › Fig. S2.tif]

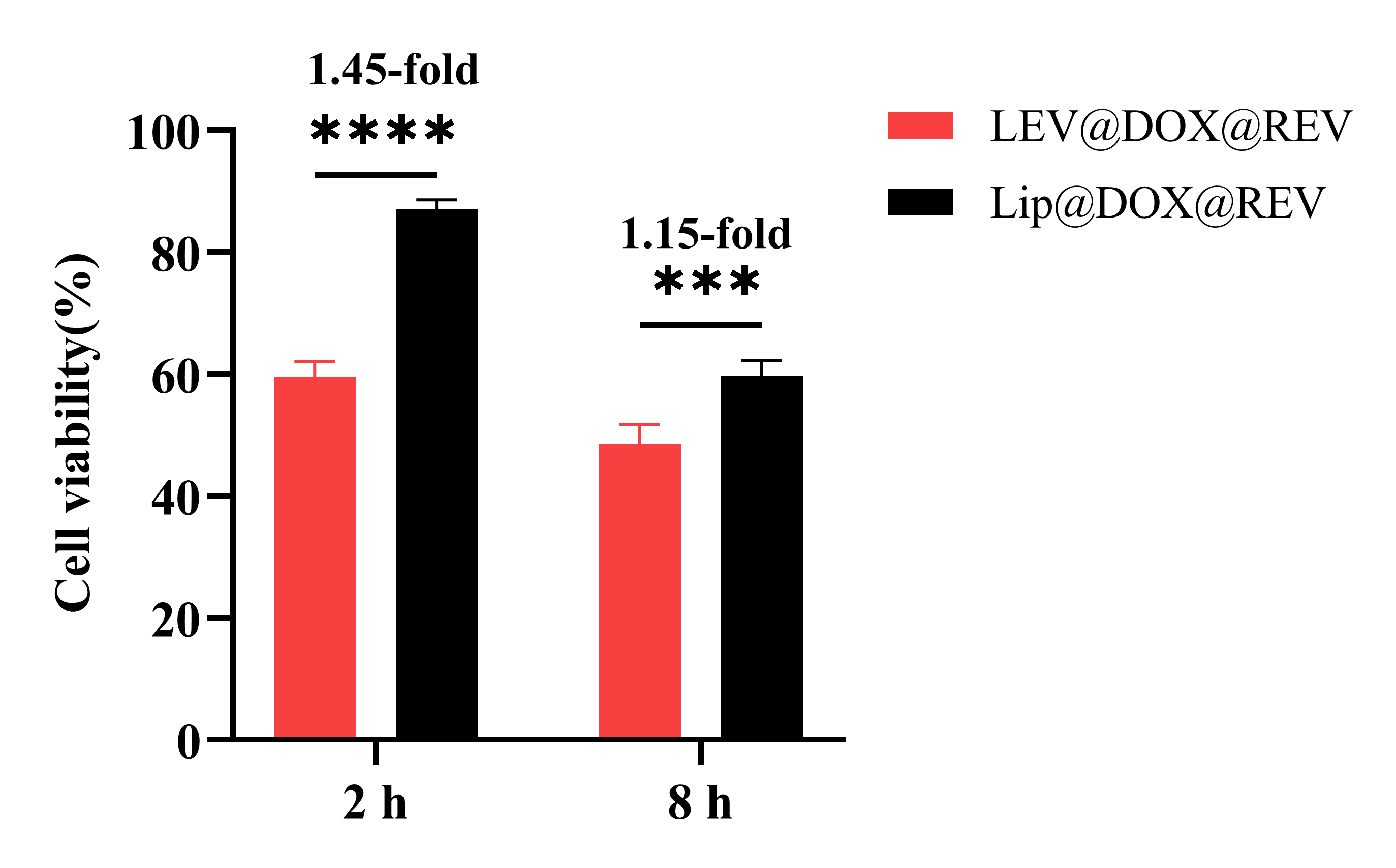

Supplement: Supplementary 1 — Figs. S1 to S6 Tables S1 and S2 [file bmr.0038.f1.zip › Fig. S4.tif]

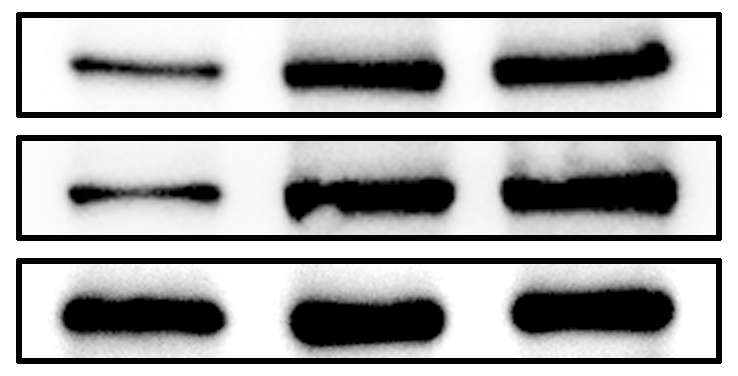

Supplement: Supplementary 1 — Figs. S1 to S6 Tables S1 and S2 [file bmr.0038.f1.zip › Fig. S5.tif]

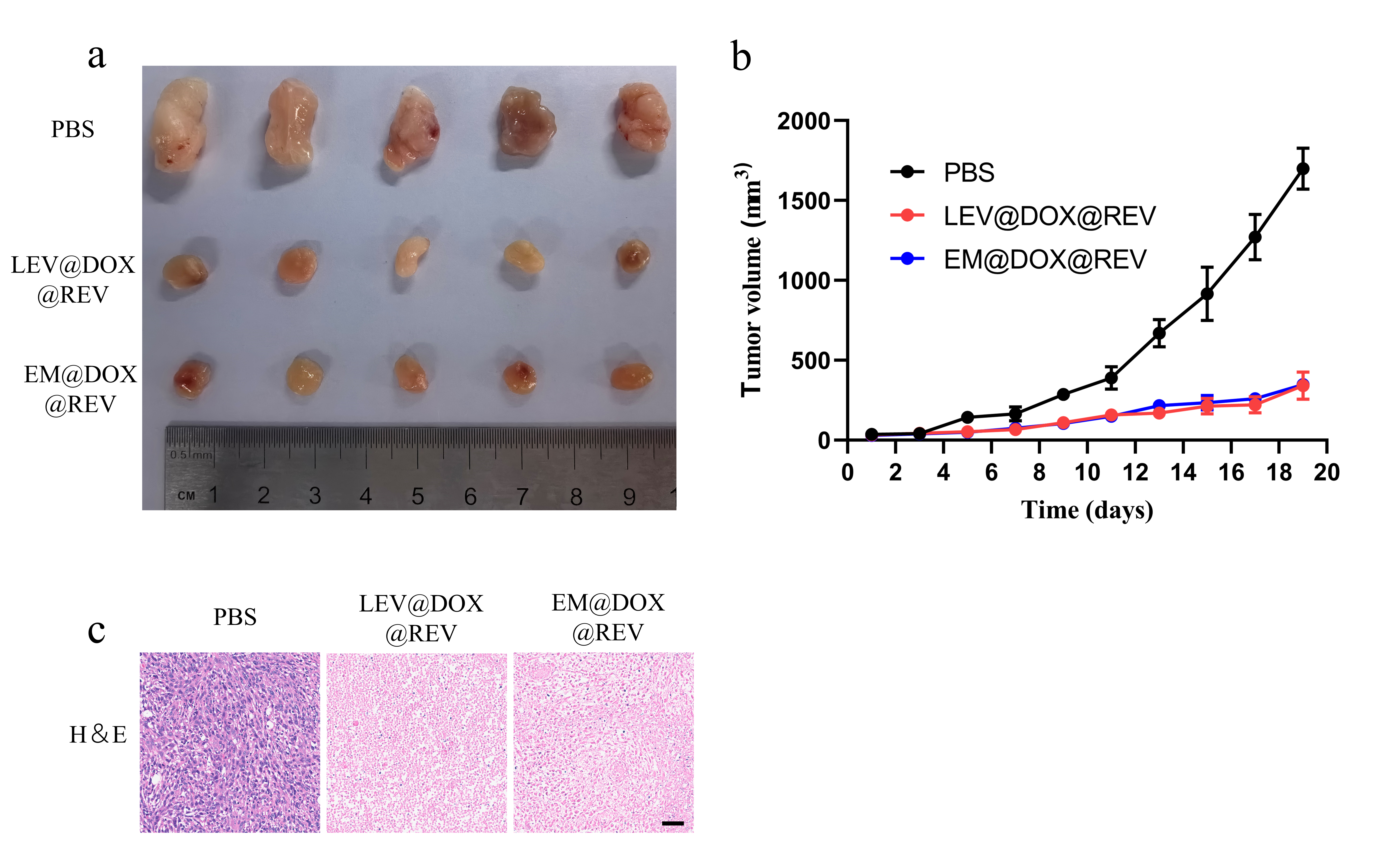

Supplement: Supplementary 1 — Figs. S1 to S6 Tables S1 and S2 [file bmr.0038.f1.zip › Fig. S6.TIF]
